# BIO-GATS: A Tool for Automated GPCR Template Selection Through a Biophysical Approach for Homology Modeling

**DOI:** 10.3389/fmolb.2021.617176

**Published:** 2021-04-07

**Authors:** Amara Jabeen, Ramya Vijayram, Shoba Ranganathan

**Affiliations:** ^1^Department of Molecular Sciences, Macquarie University, Sydney, NSW, Australia; ^2^Department of Biotechnology, Bhupat and Jyoti Mehta School of Biosciences, Indian Institute of Technology Madras, Chennai, India

**Keywords:** biophysical approach, hydrophobicity correspondence, template selection, homology modeling, GPCR, olfactory receptor, automated tool

## Abstract

G protein-coupled receptors (GPCRs) are the largest family of membrane proteins with more than 800 members. GPCRs are involved in numerous physiological functions within the human body and are the target of more than 30% of the United States Food and Drug Administration (FDA) approved drugs. At present, over 400 experimental GPCR structures are available in the Protein Data Bank (PDB) representing 76 unique receptors. The absence of an experimental structure for the majority of GPCRs demand homology models for structure-based drug discovery workflows. The generation of good homology models requires appropriate templates. The commonly used methods for template selection are based on sequence identity. However, there exists low sequence identity among the GPCRs. Sequences with similar patterns of hydrophobic residues are often structural homologs, even with low sequence identity. Extending this, we propose a biophysical approach for template selection based principally on hydrophobicity correspondence between the target and the template. Our approach takes into consideration other relevant parameters, including resolution, similarity within the orthosteric binding pocket of GPCRs, and structure completeness, for template selection. The proposed method was implemented in the form of a free tool called Bio-GATS, to provide the user with easy selection of the appropriate template for a query GPCR sequence. Bio-GATS was successfully validated with recent published benchmarking datasets. An application to an olfactory receptor to select an appropriate template has also been provided as a case study.

## Introduction

The three-dimensional (3-D) structure of the proteins is important for deciphering its biological function and gaining mechanistic insights of biological events. Analyzing the relationship between sequence, structure, and function between proteins might help in transferring functional annotation between proteins. Cyrus Chothia’s contribution in incorporating computational approaches for a sequence-structure relationship, such as the development of Structural Classification of Proteins (SCOP) database (Lo Conte et al., 2000), has opened up new avenues for structural bioinformatics. The hierarchical division of proteins into classes, folds, superfamilies, and families based on structural and functional similarities by SCOP has enabled linking of known protein structures with homologous sequences lacking a known structure. Distant homologies can also be tracked through the SCOP database (Redfern et al., 2008). The use of homolog structures for generating the structural model of a protein lacking experimental structure forms the basis of homology modeling. The success of the homology model is greatly determined by the selected template and the alignment generated between the target and the template (Wallner and Elofsson, 2005; Haddad et al., 2020). In this article, we have developed a graphical user interface for selecting suitable templates for GPCRs. Our biophysical method for GPCR template selection is based primarily on hydrophobic correspondence (HC) between the target and the template, inspired by the work of Cyrus Chothia on the conceptual methods for hydrophobicity determination (Chothia, 1976).

G protein-coupled receptors, also known as seven transmembrane (TM) domain receptors, constitute the largest family of cell surface receptors with above 800 members in humans. All GPCRs share a common architecture of seven TM helices connected through three extracellular (ECL 1–3) and three intracellular (ICL 1–3) loops with an extracellular amino (N-) terminus and intracellular carboxyl (C-) terminus (Miyagi et al., 2020). The most common classification system used for GPCRs is based on sequence and functional similarities. This schema classifies GPCRs into six classes, *viz.* class A (rhodopsin-like family), class B (secretin family), class C (metabotropic glutamate family), class D (fungal mating pheromone receptors), class E (cyclic adenosine monophosphate or cAMP receptors), and class F (frizzled/smoothened receptors). All classes of GPCRs govern myriad functionalities within the human body, ranging from sensory perception (smell, taste, vision) to neurotransmission, metabolism, immune response, blood pressure regulation, and cognition (Hu et al., 2017). GPCRs recognize diverse ligands including peptides, hormones, odorants, tastants, vitamins, photons, ions, and metabolites, among others (Wacker et al., 2017). The extracellular ligands bind to the inactive GPCRs and bring about a conformational change to the helical bundle, which in turn activates intracellular transducers such as G-proteins, or β-arrestins. The intracellular transducers are connected to the helical bundle through ICL3. Therefore, GPCRs exhibit multiple conformational states, with the active and inactive states being the predominant ones (Miyagi et al., 2020).

Dysfunction of GPCR signaling leads to pathological conditions within the human body, making GPCRs the largest druggable protein family. More than 34% of FDA approved drugs target GPCRs (Saikia et al., 2019). Currently, only ∼15% of the GPCRs are targeted. This under-representation is mainly due to the absence of known ligands for more than 30% of non-olfactory GPCRs (Insel et al., 2019). Virtual ligand screening coupled with experimentation has resulted in the discovery of novel ligands for numerous GPCRs (Congreve et al., 2020). Both ligand-based virtual screening (LBVS), as well as structure-based virtual screening (SBVs), have been used in finding novel ligands for GPCRs. LBVS can only be applied to the receptors having known ligands. Machine learning-based methods for LBVS are becoming popular for expanding the ligand set of the receptor with a large number of known ligands (Butkiewicz et al., 2019; Jabeen and Ranganathan, 2019). SBVS has also been used to find novel ligands for GPCRs (Congreve et al., 2020) but unfortunately, only 91 GPCRs have experimentally resolved structures to date, according to GPCRdb statistics (Munk et al., 2019) (as of 05.01.2021) with over 500 structures deposited in the Protein Data Bank (PDB) (Berman et al., 2000). This sequence to structure gap is mainly because of the challenges associated with structure determination of GPCRs (Baker et al., 2017; Jabeen et al., 2019a). Among the challenges are difficulties in heterologous expression, lower stability, maintaining the structural integrity by embedding into the membrane-like environment, and the existence of multiple conformations (Miyagi et al., 2020). The booming period for GPCR structural biology started in 2000 when the first GPCR structure (bovine rhodopsin) was resolved (Palczewski et al., 2000). Due to continuous improvement in structural biology methods, experimentally resolved GPCR structures are increasing but they are still under-represented compared to soluble, globular proteins. Experimental structures are now available for all classes except E (Munk et al., 2019). Most of the experimentally resolved structures belong to GPCR class A. Consequently, most of the available drugs in the market target class A receptors (Basith et al., 2018).

Homology modeling could be used for structure-based drug design (SBDD), in the absence of an experimental structure, as it is more reliable than *ab initio* modeling (Nikolaev et al., 2018). To assess the accuracy of GPCR structural model predictions, community-wide GPCR Dock competitions are conducted. Scientific research groups from all over the world are given the GPCR target sequences for blind structure prediction, with undisclosed 3D structures. The predicted models along with their atomistic interactions with pharmaceutically important small molecules, are then ranked based on the experimentally resolved structures (Kufareva et al., 2014). These competitions have shown that homology models are able to impart valuable insights into receptor-ligand interactions, especially when sequence identity between target and the template exceeds 35% (Alfonso-Prieto et al., 2019). In fact, ligand screening against dopamine D_3_ receptor was conducted initially using a homology model and provided results comparable to the experimental receptor structure (Carlsson et al., 2011).

Homology modeling of GPCRs poses several challenges, with template selection being the most prominent one. This is due to the unavailability of a close structural template for many GPCRs and limited representation of structures in active and intermediate conformations. Active structures are available for 47 receptors from classes A, B1, C, D, and F, and the structures for 20 receptors (classes A, C, and B1) are present in intermediate conformation. Also, 63 receptors are present in inactive conformation (classes A, B1, C, and F).

The accuracy of homology models is largely dependent on the choice of the template structure (Rataj et al., 2014). There are a number of servers designed specifically for GPCR homology modeling, such as GPCR-I-TASSER, GPCR Online MOdeling and DOcking server (GOMoDo) (Sandal et al., 2013), GPCR-Sequence-Structure-Feature-Extractor (SSFE) (Worth et al., 2017), GPCR-ModSim (Esguerra et al., 2016), and GPCRM (Miszta et al., 2018). The process of template selection varies among each server. GPCR-I-TASSER uses a local meta-threading server (LOMETS) (Zheng et al., 2019) to select templates for a particular GPCR. LOMETS uses eleven different threading programs (CEthreader, FFAS3D, HHpred, HHsearch, MUSTER, Neff-MUSTER, PPAS, PRC, PROSPECT2, SP3, and SparksX) to select templates for a GPCR target. GOMoDo uses the HHsearch protocol to select the template for a query GPCR sequence. The user can either use the server-generated alignment, supply their own alignment, or use a previously stored alignment for GPCR homology model building. GPCR-SSFE selects the template based on the sequence-structure profile generated by HMMER2. The webserver provides a TM-wise template suggestion. It uses 27 GPCR structures as templates. The server-generated alignment is used for model building within GPCR-SSFE. The GPCR-ModSim server uses a set of 33 structures (22 inactive, eight intermediate, and three active) and a GPCR query sequence to generate the profile alignment and then selects the suitable templates. The templates for a specific region can be also selected by the user. The server-generated alignment, as well as a manually edited alignment, can be used for model building. The GPCRM server uses sequence identity calculated by ClustalW2 for selecting the template structures. Single or multiple templates may be selected, depending upon the sequence identity between the query and the template. The server also provides the feature of selecting the template based on the user’s choice. The user can also opt for inactive or active templates. The set of templates include 63 inactive and 31 active GPCR structures.

Numerous benchmarking studies have been conducted by incorporating global and local similarity measures to select the appropriate template for GPCRs. Models based on local similarity measures have produced better results in virtual screening experiments (Castleman et al., 2019; Szwabowski et al., 2020). Multiple studies have shown that sequence identity above 30% could result in good GPCR homology models (within 3 Å) (Shahaf et al., 2016; Loo et al., 2018; Jaiteh et al., 2020). But most of the GPCRs share low sequence identity with available templates. It is also known from the literature that models based on greater sequence identity are not always the best ones and models based on distant homologs have performed well in virtual screening experiments (Rataj et al., 2014; Perry et al., 2015). Therefore, additional measures other than sequence identity must be considered for appropriate template selection. Also, a detailed inspection of all available homolog structures is essential for finding an optimal template, rather than randomly selecting a template based on the closest homolog, to generate better homology models (Kosinski et al., 2013). Sequences with similar hydrophobic patterns are often homologs, resulting in hydrophobicity being used in determining even distant homologs (Lolkema and Slotboom, 1998; Silva, 2008). The consideration of hydrophobic information for GPCR model building enables the representation of functional aspects as well (Crasto, 2010).

**FIGURE 1:**
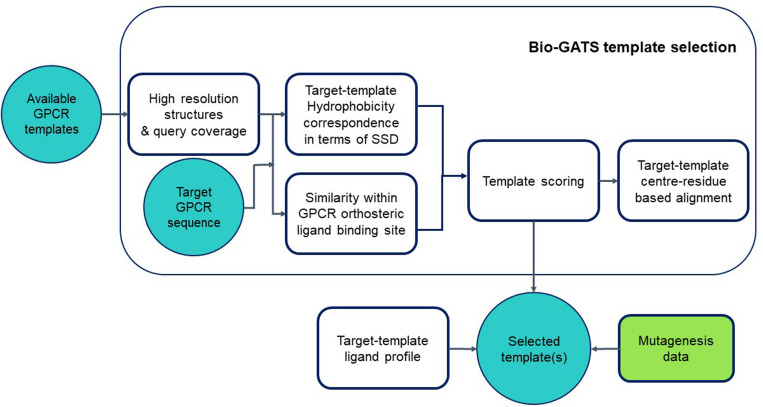
Workflow for GPCR template selection through a biophysical approach, with details of how templates are selected and the alignment is generated.

We proposed a biophysical approach recently for GPCR template selection (Jabeen and Ranganathan, 2020), which was applied to an olfactory receptor (OR), based on hydrophobicity correspondence (HC), the resolution, completeness of structures (or query coverage), and similarity between the residues within the orthosteric binding pocket for GPCRs (hotspot residues). Bio-GATS presents a GUI for template selection of GPCRs, based on this biophysical approach (Figure 1). Ligand profiles for selected templates and the target can be compared to get an optimal template. Further incorporation of mutagenesis data while refining the binding pocket of the model might help in improving the overall model.

As a case study, we have selected OR1A1, a human OR, as a query sequence. ORs are the largest superfamily of GPCRs and have no known experimental structure. Only 30 of 405 human ORs are currently known as proteins, with the rest regarded as “missing” proteins on account of insubstantial proteomic evidence (Jabeen et al., 2019a). ORs share low sequence identity with available GPCR structures. Therefore, it is challenging to get a reliable homology model for any OR. OR1A1 is ectopically expressed in gut enterochromaffin cells and proposed to be involved in serotonin release (Braun et al., 2007). Also, OR1A1 is known to be ectopically expressed in HepG2 liver cells where it is responsible for hepatic triglyceride metabolism modulation (Wu et al., 2015).

## Materials and Methods

Bio-GATS is written in Python 3 programming language (Van Rossum and Drake, 2011). The interface was built using PyQt5. The computing was performed through pandas. The numPy library was utilized for mathematical tasks. Biopython (Cock et al., 2009) was used for running BLAST (Altschul et al., 1990) locally through the command line, and for aligning the query sequence with that of the template. The HC plots were visualized using matplotlib. The hydrophobicity moment was calculated and plots were visualized using modlAMP package (Müller et al., 2017). A downloadable result summary file, from which images and data can be easily extracted, is generated in Microsoft (MS) Word format, using the docx library.

Bio-GATS requires Python, Biopython and also local BLAST to be installed locally to align sequences and then calculate the sequence identity values. Bio-GATS is linked to the GPCR dataset stored in an MS Excel file, which can be updated locally, as new GPCR structures are solved. The template selection process is divided into three steps: TM splitting and alignment, HC calculation, and finally, sequence similarity calculation among hotspot residue positions within the target and the template.

Also, a scoring matrix has been defined to rank the templates. The final score of the template is calculated based on resolution, the HC score, and binding site (or hotspot) residue similarity (BRS) score.

### GPCR Dataset

The dataset used by Bio-GATS comprises GPCR sequences, available GPCR structural templates, TM definition of each entry and structure resolution, conformation, and positions having structural information for each of the available templates (query coverage). The data for available GPCR structures were downloaded from GPCRdb. It contains 76 unique receptors and over 400 PDB entries (as of 05.08.2020). The resolution of GPCR structures varies from 1.7 to 7.7 Å. Some GPCRs are over-represented, with 52 different structures of variable resolution available for bovine rhodopsin (UniProtKB OPSD_BOVIN) followed by 49 structures for human adenosine receptor A2a (AA2AR_ HUMAN). The data for 814 GPCR sequences and their TM definitions were taken from the published GpcR Sequence-Structure (GRoSS) alignment (Cvicek et al., 2016).

### TM Splitting and Alignment

During the first step, the sequence of each TM was retrieved after splitting the sequence of both target and template according to the TM definitions taken from the GRoSS alignment. The corresponding TMs of target and template were then aligned together by tethering the center residues of each helix, as adopted by several groups (Wolf et al., 2017; Abaffy et al., 2018). The center residue for each helix is labeled as X.50 (X being the TM number), according to *Ballesteros*–*Weinstein* numbering scheme (Ballesteros and Weinstein, 1995).

### Hydrophobicity Profile Generation

The hydrophobicity profile for each helix was generated using the Eisenberg scale (Eisenberg et al., 1984), as detailed in our recent publication (Jabeen and Ranganathan, 2020) and briefly outlined here. A moving window of size 11 was set up as suggested for the identification of putative transmembrane α-helices (Wallace et al., 2004). The average value over all the residues in a window was taken and ascribed to the center residue of the window. We then measured the HC between each aligned helix of the target and the template. The HC is represented as the sum of squared differences (SSD) (eq. 1 and eq. 2):

(1) $$H_{n}=\sum_{i=n-5}^{n+5}h_{i}/11$$

(2) $$SSD=\sqrt{\sum_{n=1}^{N}{\left(H_{template,n}-H_{target,n}\right)}^{2}}$$

where *H*_*n*_ is the calculated hydrophobicity for the aligned template-target residue in the *n*th position of the alignment and *h*_*i*_ is the hydrophobicity of the *i*th residue from the Eisenberg scale. The value, is normalized by dividing with the total number of residues in a particular helix, as the SSD value is length dependent and will only be relevant if a per-residue value is considered.

### Calculating Sequence Similarity Between Hotspot Residues Known for GPCRs

We have taken the 24 traditional orthosteric ligand binding positions observed in most of the available GPCR structures. The positions are labeled according to *Ballesteros*–*Weinstein* numbering scheme and include 3.28, 3.29, 3.32, 3.33, 3.36, 3.37, 4.52, 5.39. 5.40, 5.43, 5.44, 5.47, 5.53, 6.44, 6.48, 6.51, 6.52, 6.55, 6.58, 7.31, 7.34, 7.38, 7.41, 7.42 (Chan et al., 2019). The similarities between these hotspot residues among the target-template pairs were computed using GPCRtm scoring matrix, designed specifically for GPCRs considering the compositional bias of hydrophobic TM regions (Rios et al., 2015).

### Target-Template Scoring

Each of the selected templates is scored based on two parameters: the HC-score and the BRS score (Munk et al., 2019). For each aligned helix, if the SSD per residue is between 0 and 0.1, 2 is added to the HC-score, while for SSD per residue >0.1, 1 is subtracted from the HC-score. This scheme is adapted from the BLAST match and mismatch scoring scheme and provides significant weighting for hydrophobicity. The overall HC-score is computed for each target-template pair using eq. 3,

(3) $$\mathrm{HC-score}=S_{h}=\sum_{i=1}^{7}s_{i}$$

where *S*_*h*_ is the overall hydrophobicity correspondence score ranging from helix 1 to 7, and *s*_*i*_ is the SSD per residue per helix. *S*_*b*_ is computed through GPCRtm matrix, *S*_*r*_ is the resolution score. If the resolution is ≤ 2.5 Å, the value for *S*_*r*_ is 1, otherwise it is 0. The total score *S*_*t*_ is computed by eq. 4.

(4) $$S_{t}=S_{h}+S_{b}+S_{r}$$

*S*_*h*_ can attain a maximum value of 14 while S_*b*_ may exceed 70, depending upon the score computed by GPCRtm. To avoid biases, we normalized both *S*_*h*_ and *S*_*b*_ between 0 and 1 and computed the ranking score, *S*_*rank*_ for ranking the top three templates while searching for templates, using eq. 5,

(5) $$S_{rank}=S_{h}^{n}+S_{b}^{n}+S_{r}$$

where *S*_*rank*_ is the total score between the target-template pair, $S_{h}^{n}$ is the normalized HC-score, $S_{b}^{n}$ is the normalized BRS score and *S*_*r*_ is the resolution score, retained from eq. 4.

### Homology Modeling

Bio-GATS provides a complete alignment that was used to build a 3-D structural model for SBVS using Modeller 9.18 (Webb and Sali, 2017) by a previously established protocol for GPCR homology modeling (Jabeen et al., 2019b). The sequence alignment between the target and the template can be manually adjusted using MEGA7 (Kumar et al., 2016) by tethering center residues, class A GPCR conserved motifs, and cysteine residues forming a disulphide bridge. Bio-GATS uses predicted transmembrane regions from the GRoSS sequence alignment of all known GPCRs sequences (Cvicek et al., 2016). The ligand of each template was initially copied to the 3-D model and removed later to create an empty binding pocket within the query model structure for the OR1A1 case study.

### Molecular Docking

For OR1A1, molecular docking of ligands was performed with ICM software (Abagyan et al., 1994). The binding pocket was predicted though ICMPocketFinder (An et al., 2005) and selected based on the available mutagenesis data for all ORs (Jabeen et al., 2019a).

## Results and Discussion

Bio-GATS has been tested on multiple computers, running on Linux as well as Windows platforms, and found to run successfully with the required dependencies installed. To validate our approach, we applied it to recent target-template datasets from published benchmarking studies and compared the results. We also considered representative receptors from each class (A, B, C, D, and F) with known experimental structure and built their models on the basis of templates selected by Bio-GATS. The models were then compared with the cognate experimental structures by calculating their root mean square deviation (RMSD) values. Further, we carried out a case study using an ectopically expressed olfactory receptor, OR1A1. We used the best templates from our approach, to build the models for OR1A1, which were validated by molecular docking with known ligands of the receptor, to check for retrieval of mutagenesis data important for ligand binding.

### Performance of Bio-GATS on Published Benchmarking Datasets

To assess the performance of Bio-GATS, we collated the already published target-template pairs used in benchmarking studies and/or virtual ligand screening (VLS) runs. The best benchmarked modeling pair choices, as well as pairs which did not perform well, were considered for the analysis. The performance of the templates was ranked as good or bad, in published studies, on the basis of good ligand enrichment in VLS (Perry et al., 2015; Loo et al., 2018; Jaiteh et al., 2020), local and global (RMSD) from crystal structures (Castleman et al., 2019), and both ligand enrichment and RMSD from the crystal structure (Shahaf et al., 2016). Researchers have compared varied parameters in these studies among the target-template pairs, including global sequence identity, TM-wise sequence identity, local sequence identity (identity within the binding pocket), model refinement through molecular dynamics and/or induced-fit docking, and the ligand binding site plasticity. These parameters were applied to classify templates as good or bad in their publications.

We applied our approach to these selected target-template pairs and compared the results of published studies and our approach. A total of 28 target-template pairs for nine different GPCR targets belonging to class A and published within last 5 years were considered for comparison. We calculated *S*_*t*_ for each target-template pair. All target-template pairs rankings in the benchmarking studies corresponded to the numerical *S*_*t*_ values (Table 1 and Supplementary Table 1). The top *S*_*t*_ scores for each target was ranked “good” in the benchmarking studies.

**TABLE 1:** Performance of Bio-GATS on recent published target-template pairs.

Target receptor	Template pairs	Published ranking	*S*_*t*_
hPAR2	hPAR1 [36]	Good	52
	hOPRX [36]	Good	31
	bOPSD [36]	Bad	10
h5HT7	hOPRX [34]	Good	41
	hPAR1 [34]	Bad	30
hPAR1	hOPRK [33]	Good	42
	hOPRX [33]	Good	40
	hAA2AR [33]	Bad	19
hADRB2	hOPRK [33]	Good	31
	hAA2AR [33]	Good	17
	hP2Y_12_R [33]	Bad	9
hP2Y_12_R	hPAR1 [32]	Good	26
	hOPRK [33]	Bad	15
	h5HT1B [32]	Bad	10
	hADRB2 [33]	Bad	9
hACM2	hDRD3 [32]	Good	44
	hOPRK [33]	Good	26
	hP2Y_12_R [33]	Bad	3
hFFAR1	hAT1R [32]	Good	24
	hP2Y_12_R [32]	Bad	22
h5-HT2AR	h5-HT2CR [35]	Good	71
	bOPSD [35]	Bad	20
	hAA2AR [35]	Bad	19
	hCXCR4 [35]	Bad	11
	hCNR1 [35]	Bad	9
hDRD2	hCXCR4 [35]	Good	26
	bOPSD [35]	Bad	11
	hCNR1 [35]	Bad	2

It was also evident from the collected dataset that high sequence identity does not always imply a good HC. PAR2_HUMAN shows good HC with both PAR1_HUMAN and OPRX_HUMAN, in accord with the VLS results (Perry et al., 2015), although it is closer to PAR1_HUMAN (sequence identity: 41%) than to OPRX_HUMAN (sequence identity: 28%). There are many instances where good HC is observed among the target-template pairs even the sequence identity falls below 30% (Supplementary Table 1).

Also, sequence-structure correlation is not always implied according to the published studies, for instance, the model of P2Y_12_R based on P2Y_12_R- PAR1_HUMAN pair (sequence identity: 23%) was closer to the P2Y_12_R crystal structure in comparison with the model based on the P2Y_12_R- OPRK_HUMAN pair (sequence identity: 28%) (Castleman et al., 2019). We note that the *St* scores reported here correctly rank PAR1_HUMAN as the best template over the other three templates (Table 1), without model building and VLS.

In the case of PAR2- PAR1_HUMAN and PAR2-OPSD_BOVIN pairs, although both have good HC, the hotspot residues are dissimilar, with *S*_*b*_(PAR2-OPSD_BOVIN) of −2, and *S*_*b*_(PAR2- PAR1_HUMAN) of 51. Thus, BRS comparison is a useful parameter in selecting the appropriate template for GPCRs. Overall, the *S*_*t*_ score is able to identify the best template for each of the nine target receptors in Table 1.

### Validating Bio-GATS Template Selection Through Experimentally Resolved GPCR Structures

**FIGURE 2:**
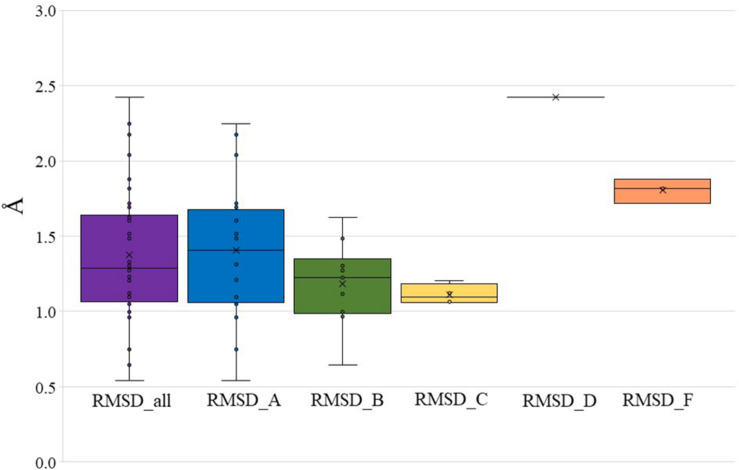
RMSD between modeled structures and experimental structures for all GPCR classes, class A, B, C, D, and F (presented in Supplementary Table 2). The boundary of the box closest to zero indicates the 25th percentile, a black line within the box marks the median, and the boundary of the box farthest from zero indicates the 75th percentile.

To further validate Bio-GATS, we selected 20 class A, 10 class B, four class C, one class D, and three class F receptors having experimentally solved structures. In all cases, the experimental structure was selected as the top ranked target template by Bio-GATS. Ignoring this top ranked structure, homology models for 38 receptors were build using Modeller (Webb and Sali, 2017) based on the second top template selected through Bio-GATS. The alignment was manually edited within loop regions through MEGA7 (Kumar et al., 2016). The generated models were compared with experimental structures through RMSD calculation for TM regions. For all models the RMSD of structurally aligned region was in the range 0.5–2.5 Å (Supplementary Table 2) as shown in Figure 2 (mean = 1.38 ± 0.43 Å, median = 1.29 Å). The interquartile range (IQR) for all classes is 0.60 Å. For individual classes, class A is showing the IQR from 0.62 Å with sample size of 20. The IQR for class B and C is 0.36 and 0.16 with sample size of 10 and 4, respectively. To date, as only one structure is available for class D, this template was selected for this receptor, although it is phylogenetically distant and therefore showing a high RMSD value. The IQR for modeled class F receptors 0.1 with sample size 3 although two of three models were built on the basis of class B templates. The results of this study on 38 representative receptors from each class are showing the utility of hydrophobicity correspondence as a measure for template selection. The median for individual classes was under 1.5 Å except for classes D and F.

Subsequently, three receptors from classes A, B, C, F, and the single class D receptor was modeled through GPCR modeling servers such as GPCR-ModSim (Esguerra et al., 2016), GoMoDo (Sandal et al., 2013), GPCRM (Miszta et al., 2018), and GPCR-SSFE (Worth et al., 2017). The RMSDs for TM regions of automated models and models constructed using Bio-GATS suggested templates were compared (Table 2). We chose to compare RMSDs of TM regions only as loop modeling and refinement within servers is a time taking process. GPCR-SSFE was only able to generate models for class A GPCRs. While GPCR-ModSim cannot accept input sequence greater than 600 residues therefore, could not model selected class C GPCRs and one class F GPCR, i.e., SMO_human. Also, for all the receptors from class A to F considered for this study, GPCR-ModSim always selected the template from class A. Of 13 GPCRs, five models built on the basis of templates selected by Bio-GATS showed minimum RMSD with experimental structure of the receptor. The four models constructed by GPCRM (CRFR1_human, GRM1_human, GRM5_human, SMO_human) were based on receptor’s own structure as a template therefore, showing the minimum RMSD (Table 2). The RMSD comparison shows the utility of our biophysical method to select the appropriate templates for all classes of GPCRs.

**TABLE 2:** The templates selected by Bio-GATS and the automated servers for representative GPCRs from each class along with RMSD values between the generated models and experimental structures for TM residues.

Receptor and PDBID	Bio-GATS		GPCRM		GPCR-SSFE		GPCR-ModSim		GoMoDo	
	Template and PDBID	RMSD (Å)	Template	RMSD (Å)	Template	RMSD (Å)	Template	RMSD (Å)	Template	RMSD (Å)
h5HT2A (6A94)	tADRB1 (4BVN)	**1.313**	tADRB1 (5F8U and 2VT4)	1.347	Many^1^	1.717	tADRB1 (2VT4)	1.427	None^6^	–
hTA2R (6IIU)	bOPSD (1U19)	2.248	hCNR1 (5TGZ) hAA2AR (5UIG)	**1.804**	Many^2^	1.975	hOPRX (4EA3)	1.948	hOPRX (4EA3)	1.911
hPE2R3 (6AK3)	hOPRM1 (5C1M)	*1.623*	h5HT2C (6BQG and 6BQH)	**1.484**	Many^3^	1.939	bOPSD (3PQR)	2.013	hOPRX (4EA3)	2.005
hCRFR1 (4K5Y)	hGLR (5EE7)	**1.626**	hCRFR1* (4Z9G)	0.966*	None^4^	–	hP2Y12 (4NTJ)	2.738	h5HT1B (4IAR)	1.853
hPACR (6P9Y)	hSCTR (6WZG)	**0.645**	hCALCR (5UZ7)	1.273	None^4^	–	bOPSD (3PQR)	2.192	None^6^	–
hSCTR (6WZG)	hCALRL (6UVA)	**1.304**	hCALCR (5UZ7)	1.466	None^4^	–	hACM2 (4MQS)	2.174	hCRFR1 (4K5Y)	1.773
hGRM1 (4OR2)	hGRM5 (6N52)	**1.122**	hGRM1* (4OR2)	0.108*	None^4^	–	None^5^	–	None^6^	–
hGRM5 (6N52)	hGRM1 (4OR2)	**1.207**	hGRM5* (5CGC, 5CGD)	0.875*	None^4^	–	None^5^	–	hOPRM1 (4DKL)	2.369
hGABR1 (6W2Y)	hGABR2 (7C7S)	**1.057**	hGRM1 (4OR2) hGRM5 (5CGC)	1.537	None^4^	–	None^5^	–	hGRM1 (4OR2)	1.492
ySTE2 (7AD3)	hGLP1R (6X19)	*2.425*	hOPRM1 (5C1M) h5HT2C (6BQH)	**2.298**	None^4^	–	hADRB2 (3SN6)	2.968	hP2Y12 (4PXZ)	2.721
hFZD4 (6BD4)	hPTH1R (6FJ3)	**1.878**	tADRB1 (5F8U, 2VTR)	2.916	None^4^	–	hPAR1 (3VW7)	–	hPAR1 (3VW7)	2.179
hFZD5 (6WW2)	hPTH1R (6FJ3)	1.817	hSMO (4O9R,4QIN)	**1.427**	None^4^	–	hADRB2 (2RH1)	–	hSMO (4JKV)	1.461
hSMO (5V56)	mSMO (6O3C)	**1.717**	hSMO (5L7I)*	0.745*	None^4^	–	None^5^	–	h5HT1B (4IAR)	1.944

The minimum RMSD values are in bold and second best values are in italics. The human GPCRs are prefixed by h, mouse by m, zebra fish by z, common turkey by t, yeast by y, and bovine by b. *self template used; RMSD values were therefore not considered. ^1^GPCR-SSFE templates: hACM4 (5DSG), hHRH1 (3RZE), hDRD3 (3PBL), hPAR2 (5NDD), h ADRB2 (2RH1), hP2Y12 (4NTJ), bOPSD (1U19), hACM3 (4U15). ^2^GPCR-SSFE templates: hPAR1 (3VW7), zLPA6 (5XSZ), hP2Y12 (4NTJ), hCXCR4 (3ODU), hAA2AR (4EIY), hPAR2 (5NDD). ^3^GPCR-SSFE templates: mOPRD1 (4EJ4), hP2Y12 (4NTJ), hCXCR4 (3ODU), sOPSD (2Z73), hCCR5 (4MBS), hHRH1 (3RZE), hPAR2 (5NDD). ^4^GPCR-SSFE does not work on non-Class A GPCRs. ^5^GPCR-ModSim does not work sequences greater than 600 residues such as hGRM1, hGRM5, hGABR1, and hSMO. ^6^GOMoDo does not work for h5HT2A, hPACR, and hGRM1.

To further extend the application of Bio-GATS we built three models each for class A and C orphans through servers as well as on the basis of Bio-GATS suggested templates. The structural alignment of automated models and manual model (based on Bio-GATS template) for GPR35_human showed the differences in modeling TM1 by GPCRSSFE and TM6 by GPCRM. For P2RY10, the model built by GoMoDo was distorted with disoriented TM1 (Supplementary Figure 1). For class C orphans, there were significant differences among all the automated and manual models as shown by structural superposition (Supplementary Figure 2) and RMSD values (Supplementary Table 3).

### Case Study on OR1A1

**FIGURE 3:**
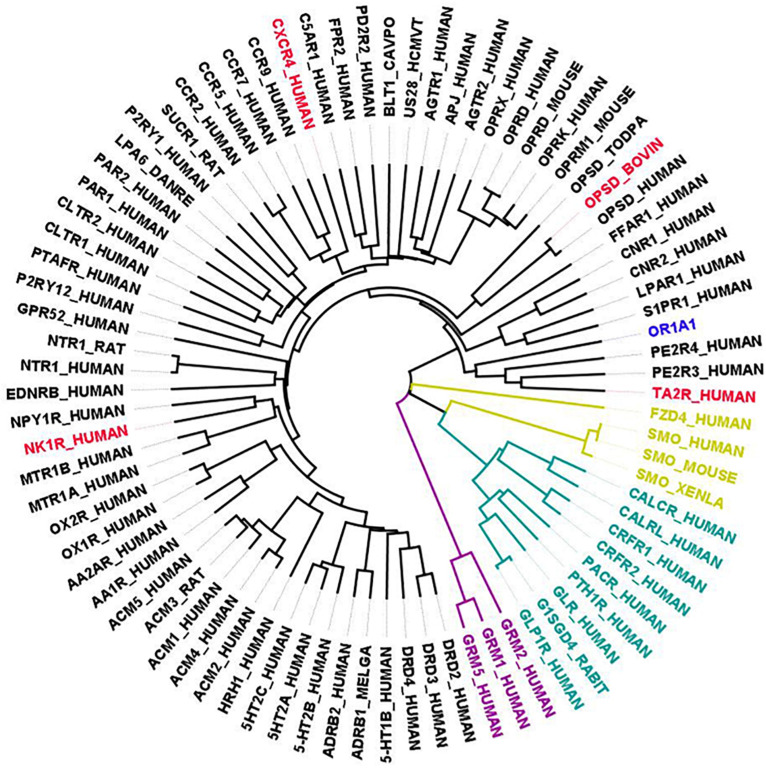
Phylogenetic tree showing all available GPCR templates are distantly related to OR1A1. The selected templates for OR1A1 are shown in red color, members having known structures for class A are in black, Class B1 are in green color, Class C are in purple, and Class F are in gold color.

Currently, there exists no close homolog for ORs as evident from the phylogenetic tree between available GPCR templates and OR1A1 (Figure 3). We used Bio-GATS to search for an optimal template for OR1A1. We selected OR1A1 as a case study because it contains the maximum mutagenesis data against six ligands among OR superfamily. The selection of templates was done on the basis of resolution (Insel et al., 2019), matching hydrophobicity profiles (*S*_*h*_), and the BRS score (Munk et al., 2019). We considered inactive structures having ≤ 2.5 Å resolution, in accord with our earlier study on OR1A2 (Jabeen and Ranganathan, 2020). The top three templates selected by Bio-GATS for OR1A1 are human NK-1 or tachykinin receptor 1, NK1R_HUMAN (PDBID: 6HLP), bovine rhodopsin, OPSD_BOVIN (PDBID: 1U19) and the human thromboxane A2 receptor, TA2R_Human (PDBID: 6IIU). We also considered one template (CXCR4_HUMAN, PDBID: 3ODU) that was showing poor HC and low BRS score with OR1A1, for comparison, from the downloadable Bio-GATS result summary table (available from Bio-GATS Github page). All four structures belong to class A GPCRs. 6HLP and 6IIU show greater than 35% sequence identity with OR1A1 (Table 3).

**TABLE 3:** Parameters used by Bio-GATS to predict top templates for OR1A1.

Rank	Template	Sequence identity (%)	Resolution (Å)	*S*_*h*_	*S*_*b*_	*S*_*r*_	*S*_*t*_	*S*_*rank*_
1	6HLP	37	2.2	11	6	1	18	2.91
2	1U19	20	2.2	8	8	1	17	2.75
3	6IIU	36	2.5	8	8	1	17	2.75
22	3ODU	25	2.5	2	−9	1	−6	1.54

*Sequence identity is listed for comparison.*

**FIGURE 4:**
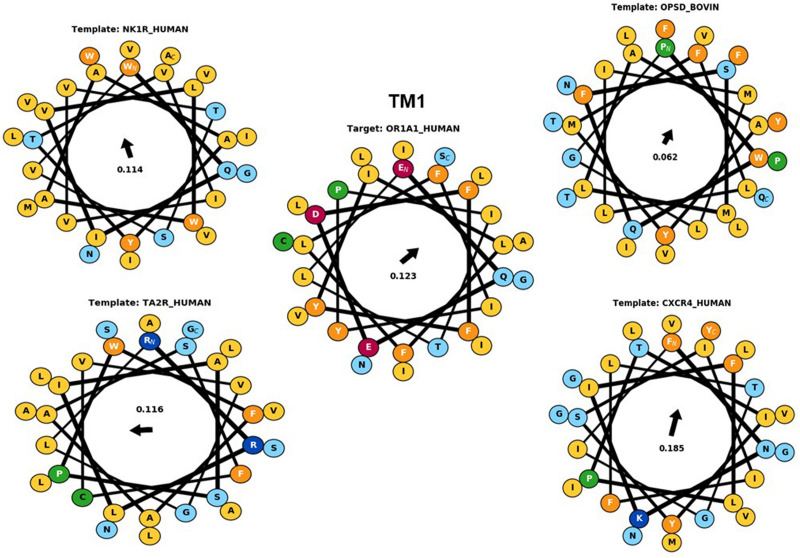
The helical wheel plots are taken from Bio-GATS for the TM1 of the target sequence (OR1A1) and the templates [NK1R_Human (6HLP), OPSD_BOVIN (1U19), TA2R_Human (6IIU), and CXCR4_Human (3ODU)]. The hydrophobic moment for OR1A1 and 1U19 are pointing in similar directions.

Hydrophobic correspondence for each TM of the top two templates 6HLP and 1U19 compared to OR1A1 are shown in Supplementary Figures 3, 4, with the other two templates to OR1A1 shown in Supplementary Figures 5, 6. All OR1A1 TMs have good HC with 6HLP TMs, except TM6. OR1A1 shows good HC with 1U19 from TM1 to TM5 but not for TM6 and TM7, while it shares good HC with 6IIU in TM1, 2, 3, 5, and 6 but not in TM4 and TM7. The OR1A1 has poor HC throughout with 3ODU except within TM1, 3, and 5. The hydrophobic moment was calculated for both the target sequence as well as the template sequences. The hydrophobic moment plots show the amphiphilic nature of the helices for the target as well as templates (TM1 in Figure 4, TM2–7 in Supplementary Figures 7–9). Amphiphilic helices are partly in the membrane and partly exposed to the aqueous phase. We used the Eisenberg scale and a window size of 11 as suitable for membrane proteins (Eisenberg et al., 1984) to calculate the hydrophobic moment of each helix. The hydrophobic moment points in the direction of maximum hydrophobicity (shown by an arrow within the hydrophobic moment plots) and it often faces the lipid surface (Liu et al., 2004). A large hydrophobic moment value shows the amphiphilicity of the helix perpendicular to its axis (Eisenberg et al., 1982). TMs 5, 6, and 7 for OR1A1 are more amphiphilic as compared to the rest of the helices. The hydrophobic moments for OR1A1 TMs 1, 2, 5, and 6 are pointing almost in the same direction as 1U19 (Figure 4 and Supplementary Figures 7–9). The incorporation of hydrophobic moment information into the structural model building is essential in the proper positioning of helices within the model (Crasto, 2010).

An example of the downloadable Bio-GATS summary file, with details of helix-wise alignment, HC comparison and hydrophobic moment results, along with the overall GRoSS alignment, is provided for the OR1A1-1U19 target-template pair in Supplementary Note 1.

For most queries, there best scoring template can be selected for analysis, and the Bio-GATS alignment can be used directly for model building and SBVS. For OR1A1, the top three templates show very similar *S*_*rank*_ scores (Table 3), suggesting that they may all be suitable for the query sequence, due to the evolutionary distance of OR1A1 (and other ORs in general) from available templates (Figure 2). Further analysis such as ligand profiling is required from our previous study on OR1A2 (Jabeen and Ranganathan, 2020), to see if all three templates are equally suitable or one is better than the other two.

**FIGURE 5:**
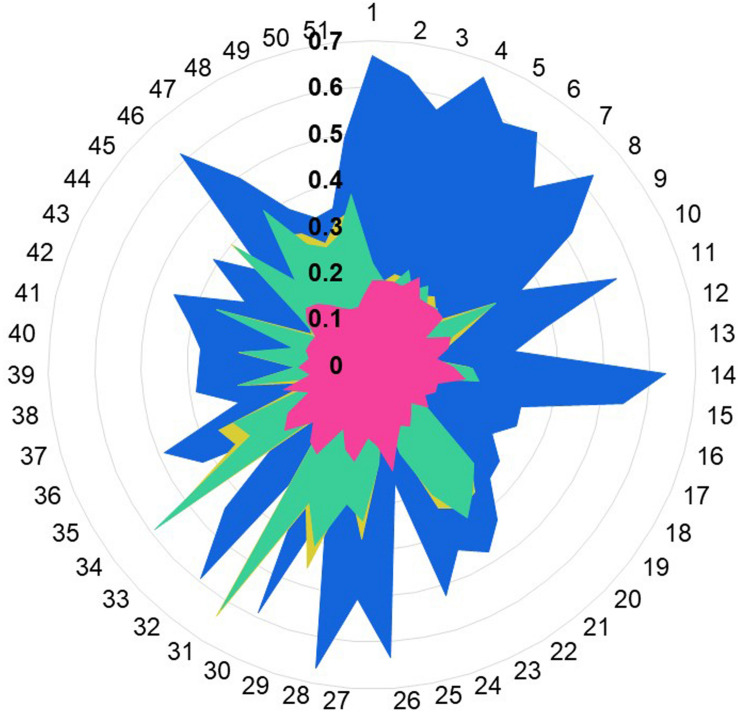
Ligand profile for OR1A1 and selected templates. The similarity of OR1A1 ligands with: retinal (from 1U19) is represented in blue color, netupitant (from 6HLP) is in gold color, ramatroban (from 6IIU) is represented in green color, and ITD (from is represented in pink color). OR1A1 ligands from 1 to 51 are listed in Supplementary Table 4. Tanimoto scores between OR1A1 ligands and the template ligands range from 0.1 to 0.7 (in bold).

We calculated the Tanimoto score between the known OR1A1 ligands and the ligand bound to the template structures, based on PubChem fingerprints computed using Knime (Berthold et al., 2009). Retinal (PubChem CID: 638015), the ligand for 1U19 (Figure 5 in blue) is more similar to the known ligands for OR1A1 followed by ramatroban (PubChem CID: 123879, Figure 4 in green) in 6IIU and netupitant (PubChem CID: 6451149, Figure 5 in gold) in 6HLP. We also compared the ligand profile for the lower scoring 3ODU and OR1A1. An isothiourea derivative, ITD (PubChem CID: 25147749, Figure 5 in pink), the ligand for 3ODU, did not match with any OR1A1 ligand (Figure 5), listed in listed in Supplementary Table 4 and is clearly not suitable for OR1A1.

The available structure for 6HLP is not complete, also the ligand profile for netupitant does not match with OR1A1 ligands. The 2nd best template 1U19 possesses a complete structure and contain a hydrophobic ligand that matches with OR1A1 ligand profile. It has the same resolution as 6HLP and S_*b*_ (8) is also better than that of 6HLP. Therefore, we selected 1U19 as a final template. To validate the Bio-GATS template selection, we built the homology model based on the suggested template (1U19) and performed molecular docking with known OR1A1 ligands having mutagenesis data and inspected whether we are able to recover the mutagenesis residues or not. For comparison, we also built a model with a template showing poor correspondence with OR1A1 in terms of *S*_*h*_, *S*_*b*_ and ligand profile.

We built models for OR1A1 based on 1U19 and 3ODU (template showing low *S*_*rank*_, and mismatched ligand profile), to differentiate between good and bad templates. We built 50 models using each template. The models with minimum Modeller objective function were selected for mutagenesis data analysis by molecular docking. Currently, OR1A1 has site-directed mutagenesis data for 13 sites for six ligands. Five positions 3.36, 3.37, 3.40, 4.56, and 5.46 are involved in (*S*)-(-)-citronellol (PubChem ID: 7793) and (*S*)-(-)-citronellal (PubChem ID: 443157) binding, 11 positions 3.34, 3.36, 3.37, 3.39, 4.53, 4.56, 5.46, 6.47, 6.48, 7.41, and 7.42 are important for (*S*)-(+)-carvone (PubChem ID: 16724) and (*R*)-(-)-carvone (PubChem ID: 439570) binding, and positions 6.48 and 6.55 are crucial for musk tibetene (PubChem ID: 67350) and musk xylene (PubChem ID: 62329) binding to OR1A1. Overall, seven positions 3.36, 3.37, 6.48, 6.55, 7.41, and 7.42 are part of the orthosteric binding site of GPCRs.

We downloaded the structures for these six ligands from PubChem and docked them to the predicted binding pocket of OR1A1, selected on available mutagenesis data. After docking (*S*)-(-)-citronellol and (*S*)-(-)-citronellal, we recovered 5/5 sites with the 1U19-based OR1A1 model but only 2/5 sites with the 3ODU-based OR1A1 model. Upon docking (*S*)-(+)-carvone and (*R*)-(-)-carvone, we were able to recover 6/11 sites with a 1U19-based model but only 3/11 sites with a 3ODU-based model. Docking musk xylene and musk tibetene into the binding pockets of OR1A1 models resulted in the recovery of both sites with a 1U19-based model and just one site using a 3ODU-model. In summary, we were able to recover maximum mutagenesis sites with the 1U19-based OR1A1 model (Supplementary Table 5). Thus, comparing the ligand profile of the target and candidate templates might be a useful measure in validating an appropriate template, in addition to the other measures. Mutagenesis data might also help in refining the predicted binding pocket of the model and has previously been incorporated to improve GPCR homology models in the literature (Ivanov et al., 2009; Perry et al., 2015).

We also used GPCR modeling servers to select the templates for OR1A1 and downloaded the generated alignment. Unfortunately, GOMoDo, and GPCR-ModSim servers did not permit submission of the query sequence therefore, results from these two servers are not included in the current study. GPCR-SSFE did not work for OR1A1 as the sequence did not match with the HMMER2 generated profile. Both GPCRM and GPCR-I-TASSER suggested AA2AR (PDBID: 3EML, resolution: 2.6 Å) as the top template. 3EML has resolution >2.5 Å and is not considered by Bio-GATS, although the high resolution AA2AR template, 5IU4 was identified as the 5th ranking template (in the result summary table, available from Bio-GATS Github page). The alignment generated by the two servers and Bio-GATS are shown in Supplementary Figures 10–12. The TM6 center residues were not aligned within the GPCRM and GPCR-I-TASSER server generated alignments but it was aligned properly by Bio-GATS. The Bio-GATS generated alignment needs manual adjustment within loop regions before proceeding to the model building step (Supplementary Figure 12).

### Bio-GATS Features

**FIGURE 6:**
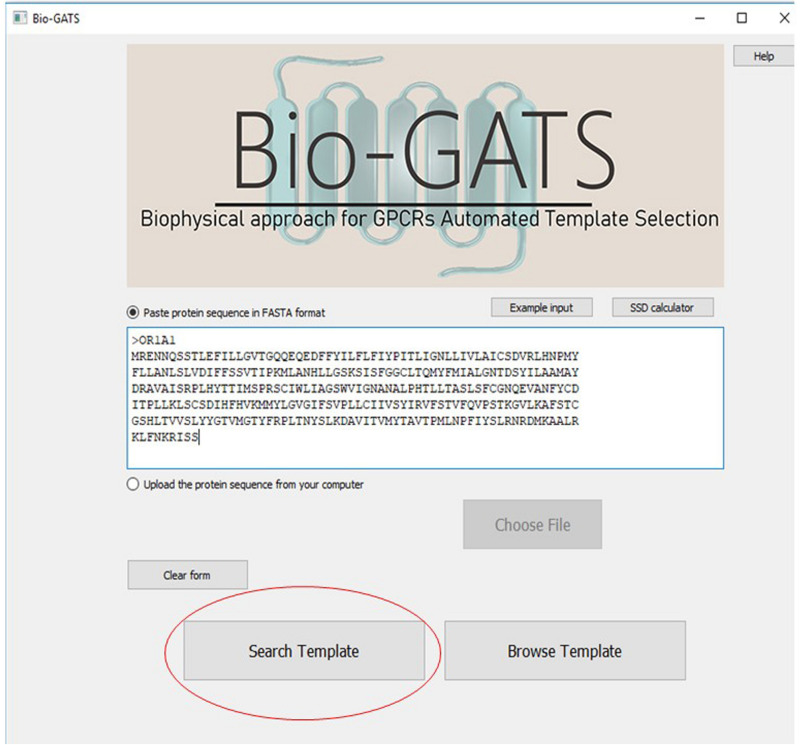
The main interface of Bio-GATS. Automated selection of templates can be done by clicking on the search template button.

Bio-GATS is connected to a local data file which contains manually curated 814 GPCR sequences, their TM definitions, PDBIDs of currently available 443 GPCR structures, their conformation, resolution, and query coverage in terms of completeness of the structure. Bio-GATS provides three main features to the users. Firstly, the user can retrieve the top three templates for the queried sequence by clicking on the search button (Figure 6).

**FIGURE 7:**
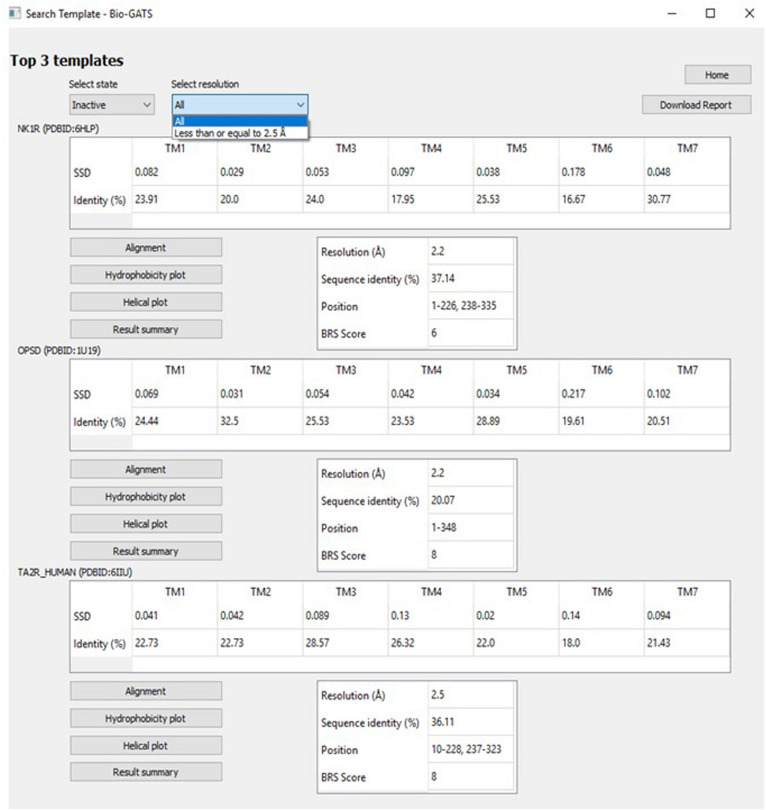
The *Search template* window with options including GPCR conformation (state) and resolution.

The top three templates are retrieved on the basis of three biophysical parameters, namely the resolution, hydrophobicity profile, and BRS score. The user can navigate among inactive, active, and intermediate conformational states as indicated in GPCRdb. The choice for selecting from a list of high resolution (≤ 2.5 Å) structures is also provided (Figure 7). For some receptors, there exist multiple PDBs as in the case of OPSD_BOVIN, with 44 PDBs available. For such a scenario, only high-quality structures were shortlisted. The quality of the structure was determined on the basis of resolution and completeness of the structure (query coverage >75%). Hence, for the search template option, high-quality structures for 54 receptors in inactive, 34 receptors in active, and 19 receptors in intermediate conformations were considered. A detailed report (shown in Supplementary Note 1) with alignments and helix-wise HC and hydrophobicity moment of each target-template pair can be downloaded for comparison and data/figure extraction. A comprehensive data table with all scoring parameters for all templates considered is also available for further analysis (examples available from Bio-GATS Github page).

For consideration of options other than resolution, HC, and BRS score for template selection, the browse functionality is available, as an advanced feature in Bio-GATS. Within this feature, the expert user might browse for the best template among the complete list of 76 receptors with 443 available PDBs. In addition to the parameters considered earlier, the browse template page provides sequence identity and TM-wise sequence identity for each template (Supplementary Figure 13). The sequence identity is calculated through a locally installed BLAST alignment. Also, all the available PDB entries, their resolution, and query coverage for each receptor can be displayed for comparison purposes (Supplementary Figure 14). The *Browse template* feature thus lists comprehensive biophysical parameters comparing the query sequence to all available templates, which might also help the user in selecting multiple templates. HC between the target and the template within the search and browse template features are based on TM definitions derived from the GRoSS alignment (Cvicek et al., 2016). For customized TM definition, a third feature, the SSD calculator, has been added to Bio-GATS, where HC is calculated based on user-defined TM definitions for both the target and the template (Supplementary Figure 15). This feature is also useful for GPCR sequences that are not present within the curated data.

The hydrophobicity plots can be visualized and downloaded for each selected target-template pair (Supplementary Figure 3). The helical wheel plots can also be shown which might help the user in identifying the helical amphiphilicities (Figure 3). Also, the center residue-based TM alignment between the target and the template can be visualized and downloaded (Supplementary Figure 16). The full-length alignment between the target and the selected template can also be downloaded in FASTA format for editing using available programs such as MEGA (Kumar et al., 2016), and AliView (Larsson, 2014), or directly building homology models through online servers such as GOMoDo (Sandal et al., 2013) or locally installed independent programs, for instance, Modeller (Webb and Sali, 2017). All these options are available from the different Bio-GATS windows. Further, a summary report (Supplementary Note 1) with the full-length alignment, TM-wise alignment, HC plots, and helical wheel plots of the target-template pair can be downloaded for detailed analysis and for use in reports and publications.

## Conclusion

The existence of low sequence identity among available GPCR structures and sequences particularly OR sequences demands additional parameters for template selection. HC, similarities within the GPCR hotspot residues and matching the target-template ligand profile might serve as additional local parameters for GPCR template selection. Further, the incorporation of mutagenesis data might be helpful in refining GPCR homology models. Bio-GATS provides a convenient and user interactive way of selecting an appropriate template for a target GPCR, based on hydrophobicity profile and hotspot residue similarity while displaying global sequence identity as well as TM sequence identity for more advanced usage. The tool provides a comprehensive biophysical comparison between a target sequence and all the available templates which might assist in selecting more than one templates, commemorating Chothia’s pioneering work in structural bioinformatics.

## Data Availability Statement

The datasets presented in this study can be found in online repositories. The names of the repository/repositories and accession number(s) can be found below: https://github.com/amara86/Bio-GATS.

## Author Contributions

AJ and SR designed the study and wrote the manuscript. AJ acquired the data. AJ and RV implemented the interface. All authors read and approved the final manuscript.

## Conflict of Interest

The authors declare that the research was conducted in the absence of any commercial or financial relationships that could be construed as a potential conflict of interest.
