# Supplementary material for: BIO-GATS: A Tool for Automated GPCR Template Selection Through a Biophysical Approach for Homology Modeling
Source: Front Mol Biosci. 2021 Apr 7;8:617176. doi: 10.3389/fmolb.2021.617176 (PMC8059640; doi:10.3389/fmolb.2021.617176)
Supplement: Supplementary file 1 [file Data_Sheet_1.pdf]

BIO-GATS: A tool for automated GPCR template selection through a biophysical approach for homology modelling

*Amara Jabeen[1], Ramya Vijayram[2], Shoba Ranganathan[1, *]*

[1] Department of Molecular Sciences, Macquarie University, Sydney, NSW 2109, Australia
[2] Department of Biotechnology, Bhupat and Jyoti Mehta School of Biosciences, Indian Institute of Technology Madras, Chennai 600036, Tamilnadu, India
* Corresponding author (shoba.ranganathan@mq.edu.au)

Supplementary Materials

Contents

Supp Table 1: Detailed parameter listing and scoring of published target-template dataset........2
Supp Table 2: RMSD calculation (TM only) between GPCR structures and models based on templates selected by Bio-GATS……………………..…………………………5
Supp Figure 1: The superimposed manual and automated models for class A orphans 6
Supp Figure 2: The superimposed manual and automated models for class C orphans 7
Supp Table 3: Template(s) selected by Bio-GATS and other servers for class A & C orphans ..8
Supp Figure 3: Helix-wise hydrophobicity correspondence between OR1A1 and 6HLP 9
Supp Figure 4: Helix-wise hydrophobicity correspondence between OR1A1 and 1U19............10
Supp Figure 5: Helix-wise hydrophobicity correspondence between OR1A1 and 6IIU 11
Supp Figure 6: Helix-wise hydrophobicity correspondence between OR1A1 and 3ODU …..…12
Supp Figure 7: Helical wheel plots from Bio-GATS for TM2 and TM3...................................13
Supp Figure 8: Helical wheel plots from Bio-GATS for TM4 and TM5...................................14
Supp Figure 9: Helical wheel plots from Bio-GATS for TM6 and TM7...................................15
Supp Note 1: Bio-GATS result summary for OPSD_BOVIN-OR1A1_HUMAN 16
Supp Table 4: Ligand profile comparison between OR1A1 and the selected templates 24
Supp Table 5: The interactions of 1U19-based and 3ODU-based OR1A1 models with known ligands of OR1A1 25
Supp Figure 10: The alignment generated by GPCR-I-TASSER 27
Supp Figure 11: The alignment generated by GPCRM 27
Supp Figure 12: The alignment generated by BIO-GATS between the query sequence (OR1A1) and the selected template, 1U19. 28
Supp Figure 13: The *Browse template* window with options 29
Supp Figure 14: The *Available PDBs* window 30
Supp Figure 15: The *SSD calculator* with customizable TM definitions. 31
Supp Figure 16: The *Show alignment* window 32
Supp References 33

Supplementary Table 1: **Detailed parameter listing and scoring of published target-template dataset as per our approach.** SI is the sequence identity, S_h is the overall hydrophobicity correspondence score ranging from helix 1 to 7, S_b is the binding site residue similarity score and S_r is the resolution score. The target-template pairs with highest value of S_t (also shown in Table 1) are in bold

**Target-template pairs**	**Res (Å)**	**Published ranking**	**SI (%)**	**SSD-TM1**	**SSD-TM2**	**SSD-TM3**	**SSD-TM4**	**SSD-TM5**	**SSD-TM6**	**SSD-TM7**	S_h	S_b	S_r	S_t
**PAR2_HUMAN- PAR1_HUMAN (PDBID: 3VW7) [1]**	**2.2**	**Good**	**41**	**0.047**	**0.008**	**0.052**	**0.048**	**0.018**	**0.054**	**0.008**	**12**	**39**	**1**	**52**
PAR2_HUMAN - OPRX_HUMAN (PDBID: 4EA3)[1]	3.0	Good	28	0.016	0.023	0.041	0.092	0.049	0.01	0.034	13	18	0	31
PAR2_HUMAN - OPSD_BOVIN (PDBID: 1U19) [1]	2.2	Bad	22	0.02	0.012	0.038	0.05	0.06	0.093	0.012	11	-2	1	10
**5HT7_HUMAN - OPRX_HUMAN (PDBID: 4EA3) [2]**	**3.0**	**Good**	**24**	**0.068**	**0.024**	**0.042**	**0.019**	**0.088**	**0.1**	**0.015**	**9**	**32**	**0**	**41**
5HT7_HUMAN- PAR1_HUMAN (PDBID: 3VW7) [2]	2.2	Bad	27	0.042	0.031	0.078	0.045	0.074	0.027	0.045	12	17	1	30
**PAR1_HUMAN - OPRK_HUMAN (PDBID: 4DJH) [3]**	**2.9**	**Good**	**27**	**0.032**	**0.024**	**0.044**	**0.042**	**0.038**	**0.058**	**0.017**	**13**	**29**	**0**	**42**
PAR1_HUMAN- OPRX_HUMAN (PDBID: 5DHG) [3]	3.0	Good	27	0.041	0.018	0.056	0.037	0.068	0.067	0.03	11	29	0	40
PAR1_HUMAN – AA2AR_HUMAN (PDBID: 3EML) [3]	2.6	Bad	21	0.028	0.056	0.105	0.02	0.046	0.028	0.027	10	9	0	19
**ADRB2_HUMAN - OPRK_HUMAN (PDBID: 4DJH) [3]**	**2.9**	**Good**	**24**	**0.063**	**0.073**	**0.012**	**0.041**	**0.117**	**0.116**	**0.073**	**5**	**26**	**0**	**31**
ADRB2_HUMAN – AA2AR_HUMAN (PDBID: 3EML)[3]	2.6	Good	30	0.096	0.012	0.046	0.023	0.033	0.022	0.099	12	5	0	17
ADRB2_HUMAN- P2Y$_{12}$R_HUMAN (PDBID: 4NTJ) [3]	2.6	Bad	21	0.05	0.053	0.134	0.059	0.047	0.113	0.11	3	6	0	9

Target-template pairs	Res (Å)	Published ranking	SI (%)	SSD-TM1	SSD-TM2	SSD-TM3	SSD-TM4	SSD-TM5	SSD-TM6	SSD-TM7	S_h	S_b	S_r	S_t
**P2Y$_{12}$R_HUMAN - PAR1_HUMAN (PDBID: 3VW7) [4]**	**2.2**	**Good**	**23**	**0.124**	**0.016**	**0.168**	**0.04**	**0.048**	**0.073**	**0.08**	**6**	**20**	**1**	**27**
P2Y$_{12}$R_HUMAN- OPRK_HUMAN (PDBID: 4DJH) [3]	2.9	Bad	28	0.095	0.043	0.101	0.079	0.062	0.154	0.051	4	11	0	15
P2Y$_{12}$R_HUMAN - 5HT1B_HUMAN (PDBID: 4IAQ) [4]	2.8	Bad	24	0.05	0.034	0.14	0.066	0.04	0.116	0.12	2	8	0	10
P2Y$_{12}$R_HUMAN- ADRB2_HUMAN (PDBID: 2RH1) [3]	2.4	Bad	21	0.05	0.053	0.134	0.059	0.047	0.113	0.11	2	6	1	9
**ACM2_HUMAN- DRD3_HUMAN (PDBID: 3PBL) [4]**	**2.9**	**Good**	**26**	**0.11**	**0.011**	**0.037**	**0.032**	**0.035**	**0.058**	**0.034**	**10**	**34**	**0**	**44**
ACM2_HUMAN- OPRK_HUMAN (PDBID: 4DJH) [3]	2.9	Good	28	0.025	0.042	0.017	0.039	0.138	0.032	0.01	11	15	0	26
ACM2_HUMAN - P2Y$_{12}$R_HUMAN (PDBID: 4NTJ) [3]	2.6	Bad	23	0.059	0.05	0.122	0.102	0.037	0.101	0.077	2	1	0	3
**FFAR1_HUMAN - AT1R_HUMAN (PDBID: 4YAY) [4]**	**2.9**	**Good**	**22**	**0.17**	**0.053**	**0.058**	**0.036**	**0.025**	**0.047**	**0.082**	**8**	**16**	**0**	**24**
FFAR1_HUMAN - P2Y$_{12}$R_HUMAN (PDBID: 4PY0) [4]	3.1	Bad	27	0.106	0.033	0.175	0.069	0.044	0.072	0.032	6	16	0	22
**5HT2AR_HUMAN-5HT2CR_HUMAN (PDBID: 6BQH) [5]**	**2.7**	**Good**	**55**	**0.02**	**0.008**	**0.01**	**0.005**	**0.008**	**0.012**	**0.053**	**13**	**58**	**0**	**71**
5HT2AR_HUMAN - OPSD_BOVIN (PDBID: 1F88) [5]	2.8	Bad	20	0.062	0.025	0.041	0.055	0.039	0.014	0.03	12	8	0	20
5-HT2AR_HUMAN– AA2AR_HUMAN(PDBID: 4EIY) [5]	1.8	Bad	26	0.109	0.013	0.033	0.038	0.015	0.016	0.03	11	7	1	19
5HT2AR_HUMAN - CXCR4_HUMAN (PDBID: 3ODU) [5]	2.5	Bad	21	0.023	0.148	0.035	0.02	0.024	0.065	0.091	9	1	1	11

**Target-template pairs**	**Res (Å)**	**Published ranking**	**SI (%)**	**SSD-TM1**	**SSD-TM2**	**SSD-TM3**	**SSD-TM4**	**SSD-TM5**	**SSD-TM6**	**SSD-TM7**	S_h	S_b	S_r	S_t
5HT2AR_HUMAN -CNR1_HUMAN (PDBID: 5U09) [5]	2.6	Bad	27	0.061	0.03	0.085	0.044	0.033	0.032	0.049	12	-3	0	9
**DRD2_HUMAN -CXCR4_HUMAN (PDBID: 3ODU) [5]**	**2.5**	**Good**	**29**	**0.063**	**0.19**	**0.034**	**0.039**	**0.09**	**0.036**	**0.037**	**9**	**16**	**1**	**26**
DRD2_HUMAN - OPSD_BOVIN (PDBID: 1F88) [5]	2.8	Bad	22	0.102	0.028	0.058	0.096	0.118	0.054	0.021	5	6	0	11
DRD2_HUMAN –CNR1_HUMAN (PDBID: 5U09) [5]	2.6	Bad	25	0.132	0.053	0.044	0.069	0.142	0.022	0.036	6	-4	0	2

Supplementary Table 2: RMSD calculation (TM only) between GPCR structures and models based on templates selected by Bio-GATS. Human GPCRs are prefixed by h, mouse by m, yeast by y, turkey by t and bovine by b.

Class	Receptor	PDBID	State	Template selected by Bio-GATS with PDBID	RMSD (Å)
A	h5HT2A	6A94	inactive	tADRB1 (4BVN)	1.56
	h5HT2C	6BQH	inactive	hADRB2 (2RH1)	1.327
	hAA1AR	5UEN	inactive	hAA2AR (5IU4)	1.192
	hAA2AR	5IU4	inactive	tADRB1 (4BVN)	2.737
	hACM1	5CXV	inactive	hACM5 (6OL9)	2.50
	hACM2	5ZKC	inactive	hACM5 (6OL9)	1.203
	hADRB2	2RH1	inactive	tADRB1 (4BVN)	0.963
	hAGTR1	4YAY	inactive	hCCR7 (6QZH)	1.897
	hCCR2	6GPX	inactive	hCCR5 (5UIW)	1.243
	hCNR2	6KPC	inactive	hCCR7 (6QZH)	1.859
	hDRD2	6CM4	inactive	tADRB1 (4BVN)	1.714
	hDRD3	3PBL	inactive	tADRB1 (4BVN)	1.519
	hHRH1	3RZE	inactive	hACM5 (6OL9)	1.478
	hOPRD	4EJ4	inactive	hOX1R (6TOS)	1.836
	hOX2R	5WQC	inactive	hOX1R (6TOS)	0.844
	hS1PR1	3V2Y	inactive	hCCR7 (6QZH)	2.032
	hTA2R	6IIU	inactive	bOPSD (1U19)	2.342
	hPE2R3	6AK3	active	bOPSD (4X1H)	1.705
	hPTAFR	5ZKP	active	mOPRM1 (5C1M)	2.376
	hP2Y12	4PXZ	Intermediate	hEDNRB (6IGK)	2.254
B	hCRFR1	4K5Y	inactive	hGLR (5EE7)	1.753
	hGLP1R	5VEW	inactive	hGLR (5EE7)	1.349
	hGLR	5EE7	inactive	hPTH1R (6FJ3)	1.551
	hPTH1R	6FJ3	inactive	hGLR (5EE7)	1.614
	hCALRL	6UVA	active	hSCTR (6WZG)	1.539
	hCRFR2	6PB1	active	hCALRL (6UVA)	1.512
	hGHRHR	7CZ5	active	hSCTR (6WZG)	1.315
	hPACR	6P9Y	active	hSCTR (6WZG)	1.004
	hSCTR	6WZG	active	hCALRL (6UVA)	1.633
	hVIPR1	6VN7	active	hSCTR (6WZG)	1.133
C	hGABR1	6W2Y	inactive	hGABR2 (7C7S)	1.969
	hGABR2	7C7S	inactive	hGABR1 (6W2Y)	2.289
	hGRM1	4OR2	inactive	hGRM5 (6N52)	1.294
	hGRM5	6N52	inactive	hGRM1 (4OR2)	1.641
D	ySTE2	7AD3	active	hGLP1R(6X19)	2.416
F	hFZD4	6BD4	inactive	hPTH1R (6FJ3)	2.005
	hFZD5	6WW2	inactive	hPTH1R (6FJ3)	1.969
	hSMO	5V56	inactive	mSMO (6O3C)	1.986

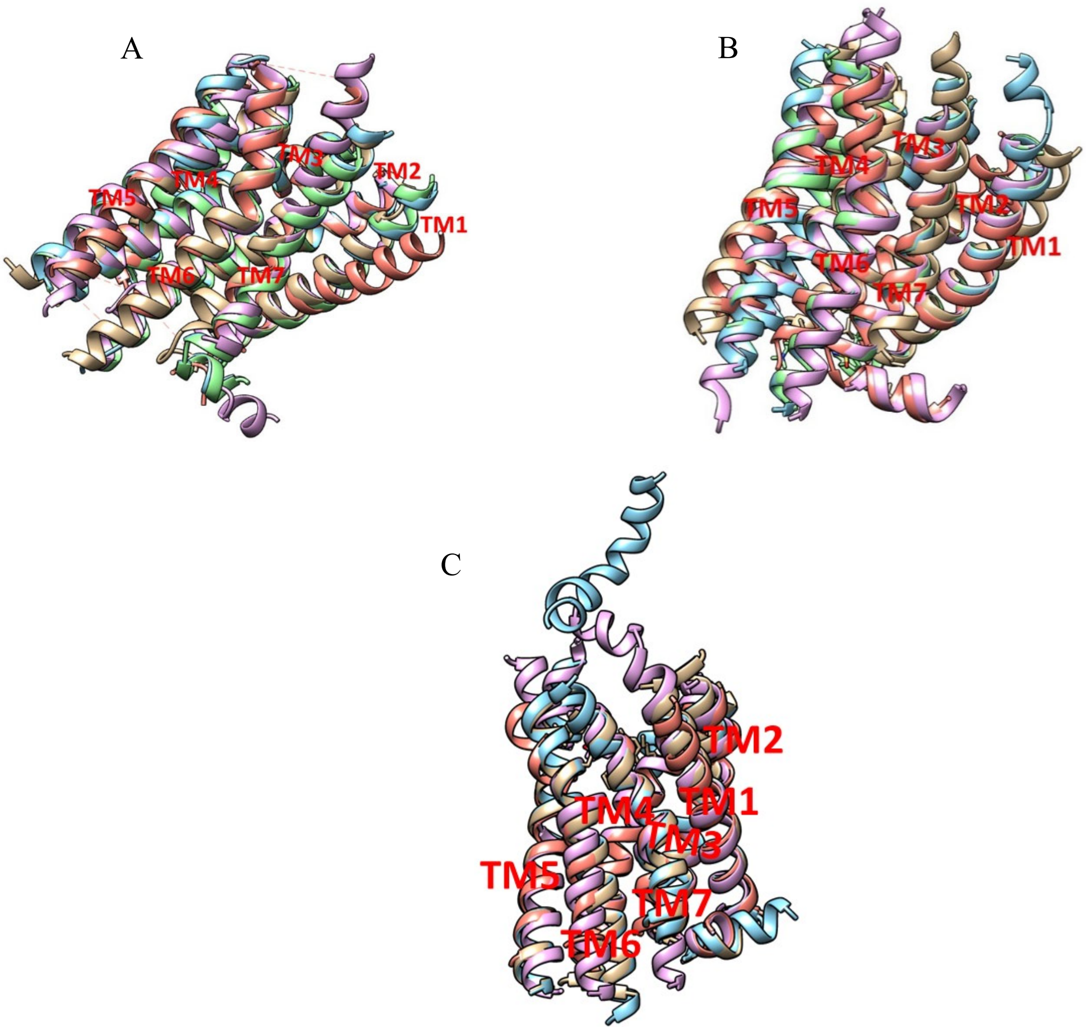

Supplementary Figure 1: The superimposed manual and automated models for class A orphans:(A) GPR35, (B) P2RY8, and (C) P2RY10. The manual model generated on the basis of Bio-GATS template is shown in gold color, GPCRM model is shown in pink color, GPCR-SSFE model is shown in rust color, GPCR-modsim model is shown in green color, and GoMoDo model is shown in blue color. Template details are available in Supplementary Table 2.

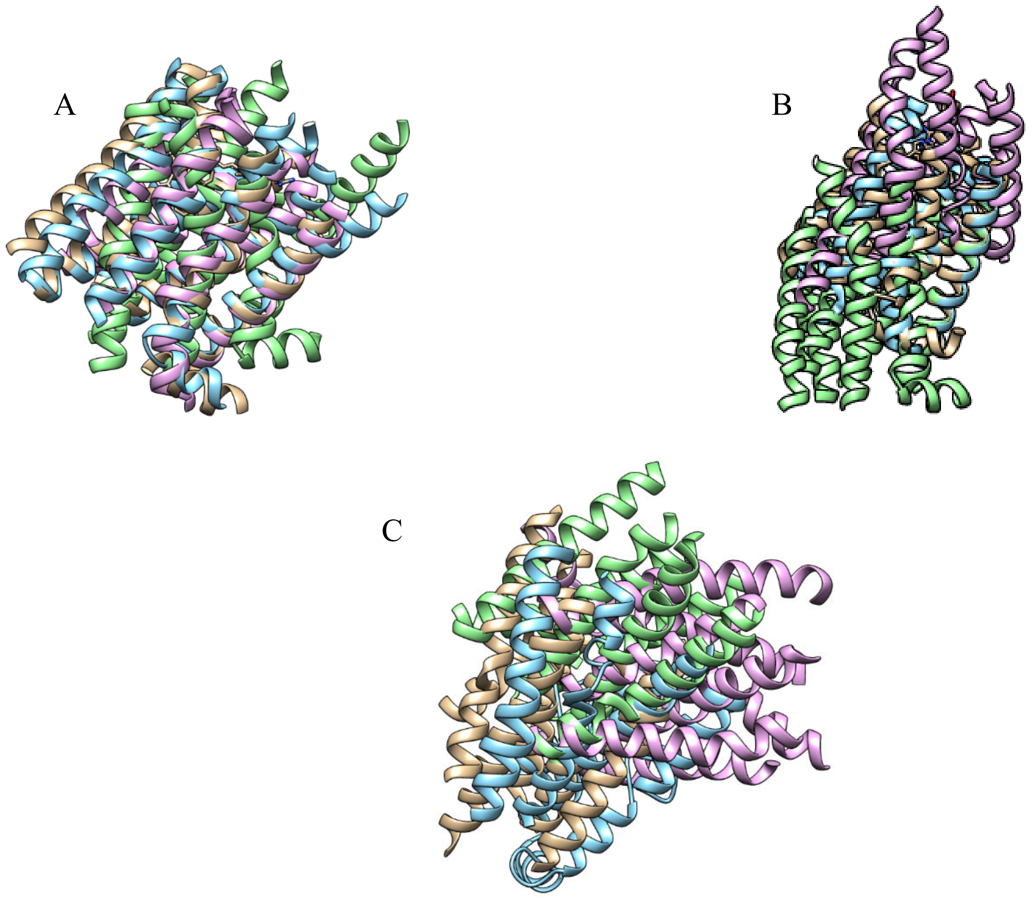

Supplementary Figure 2: The superimposed manual and automated models for class C orphans: (A) GPC5C, (B) GPC5D, and (C) RAI3. The manual model generated on the basis of Bio-GATS template is shown in gold color, GPCRM model is shown in pink color, GPCR-modsim model is shown in green color, and GoMoDo model is shown in blue color. Template details are available in Supplementary Table 2.

Supplementary Table 3: Template(s) selected by Bio-GATS and other servers for class A & C orphans with RMSD values calculated from structural alignment of models generated with the manual model. The human GPCRs are prefixed by h, mouse by m, and zebra fish by z.

Receptor	Selected template(s) with PDBID					
	Bio-GATS	GPCRM	GPCR-SSFE	GPCR-modsim	GoMoDo	RMSD (Å)
GPR35_Human (Class A-orphan)	5UIW (hCCR5)	6B73 (hOPRK), 4XNW (hP2RY1)	Many[1]	4EA3 (hOPRX)	4EA3 (hOPRX)	1.58
P2RY8_Human (Class A-orphan)	6QZH (hCCR7)	5NDD (hPAR2), 4DJH (hOPRK)	Many[2]	3VW7 (hPAR1)	3VW7 (hPAR1)	1.71
P2Y10_Human (Class A-orphan)	4N6H (hOPRD)	5NDD (hPAR2), 4XNW (hP2RY1)	Many[3]	None[5]	4N6H (hOPRD)	1.50
GPC5C_Human (Class C-orphan)	3KS9 (hGRM2)	4OR2 (hGRM1), 5CGC (hGRM5)	None[4]	2RH1 (hADRB2)	4IAR (h5HT1B)	2.98
GPC5D_Human (Class C-orphan)	5IU4 hAA2AR	4PY0 (hP2RY12) 6B73 (hOPRK)	None[4]	4DJH (hOPRK)	4OR2 (hGRM1)	2.60
RAI3 (Class C-orphan)	5UIW (hCCR5)	5UIG, 4EIY (hAA2AR)	None[4]	3UON (hACM2)	4OR2 (hGRM1)	3.62

[1]GPCR-SSFE templates: hOPRK1 (4DJH), hOPRL1 (4EA3), hCCR5 (4MBS), hCXCR4 (3ODU), mOPRD1 (4EJ4), zLPA6 (5XSZ)
[2]GPCR-SSFE templates: hPAR1 (3VW7), hDRD3 (3PBL), hOPRK1 (4DJH), hP2RY1_Human(4XNV), hCCR5 (4MBS), hPAR2 (5NDD)
[3]GPCR-SSFE templates: hOPRK1 (4DJH), hPAR2 (5NDD), zLPA6 (5XSZ), hP2Y12 (4NTJ)
[4]GPCR-SSFE does not work on non-Class A GPCRs.
[5]GPCR-modsim does not work for hP2Y10.

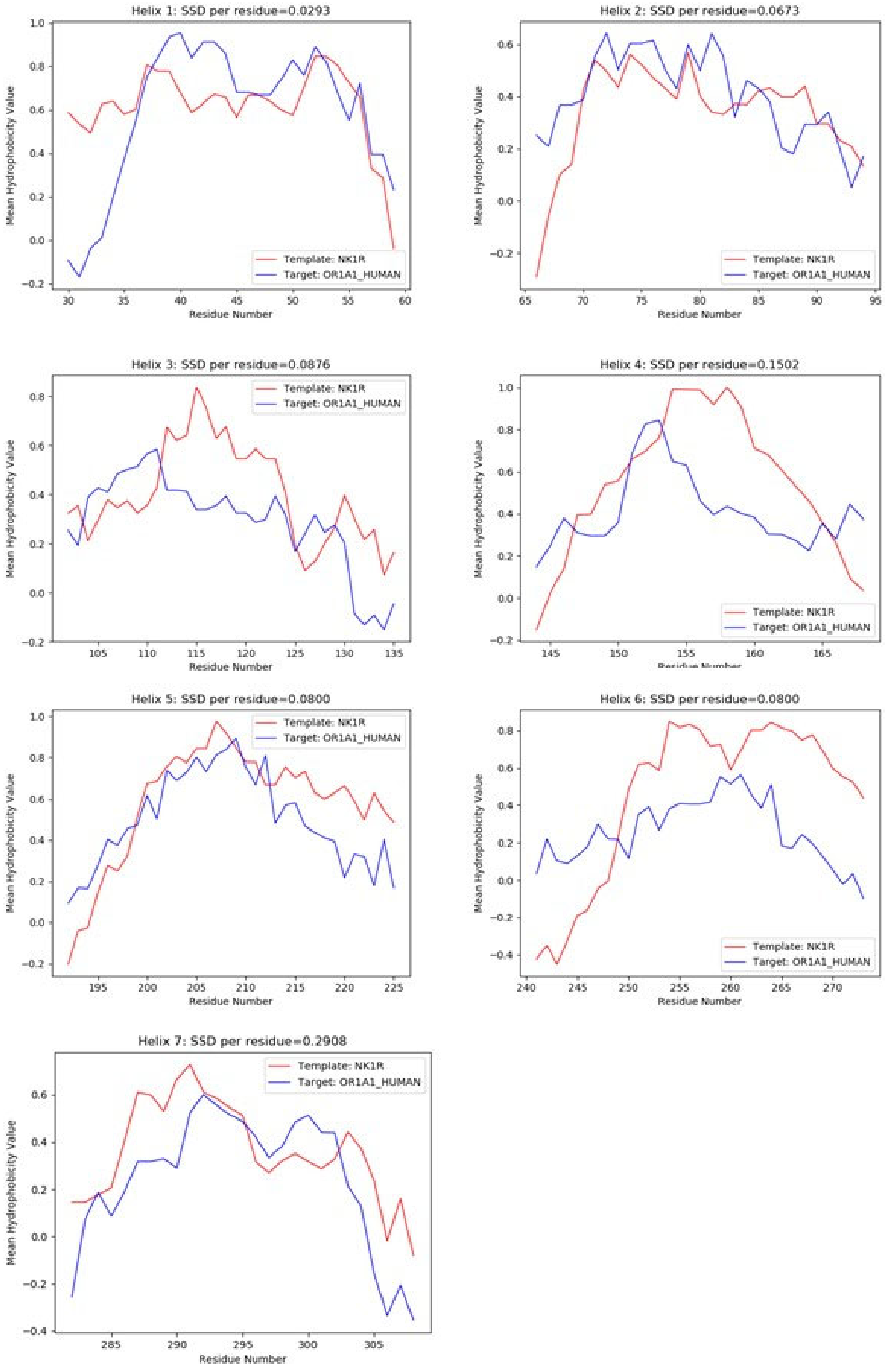

Supplementary Figure 3: **Helix-wise hydrophobicity correspondence between OR1A1 and 6HLP** (top template selected by Bio-GATS). Images are taken from Bio-GATS.

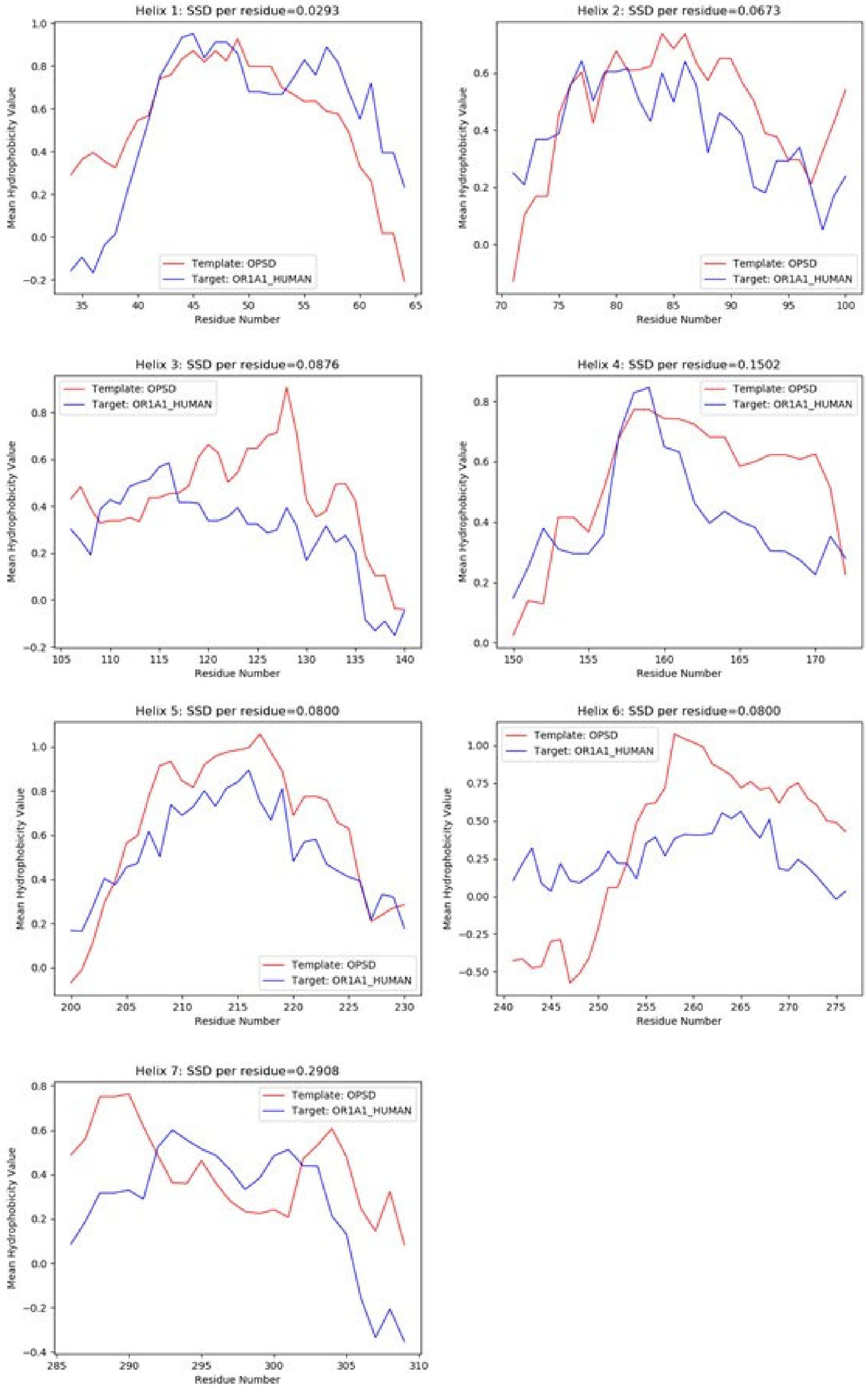

Supplementary Figure 4: **Helix-wise hydrophobicity correspondence between OR1A1 and 1U19** (the 2nd best template by Bio-GATS).

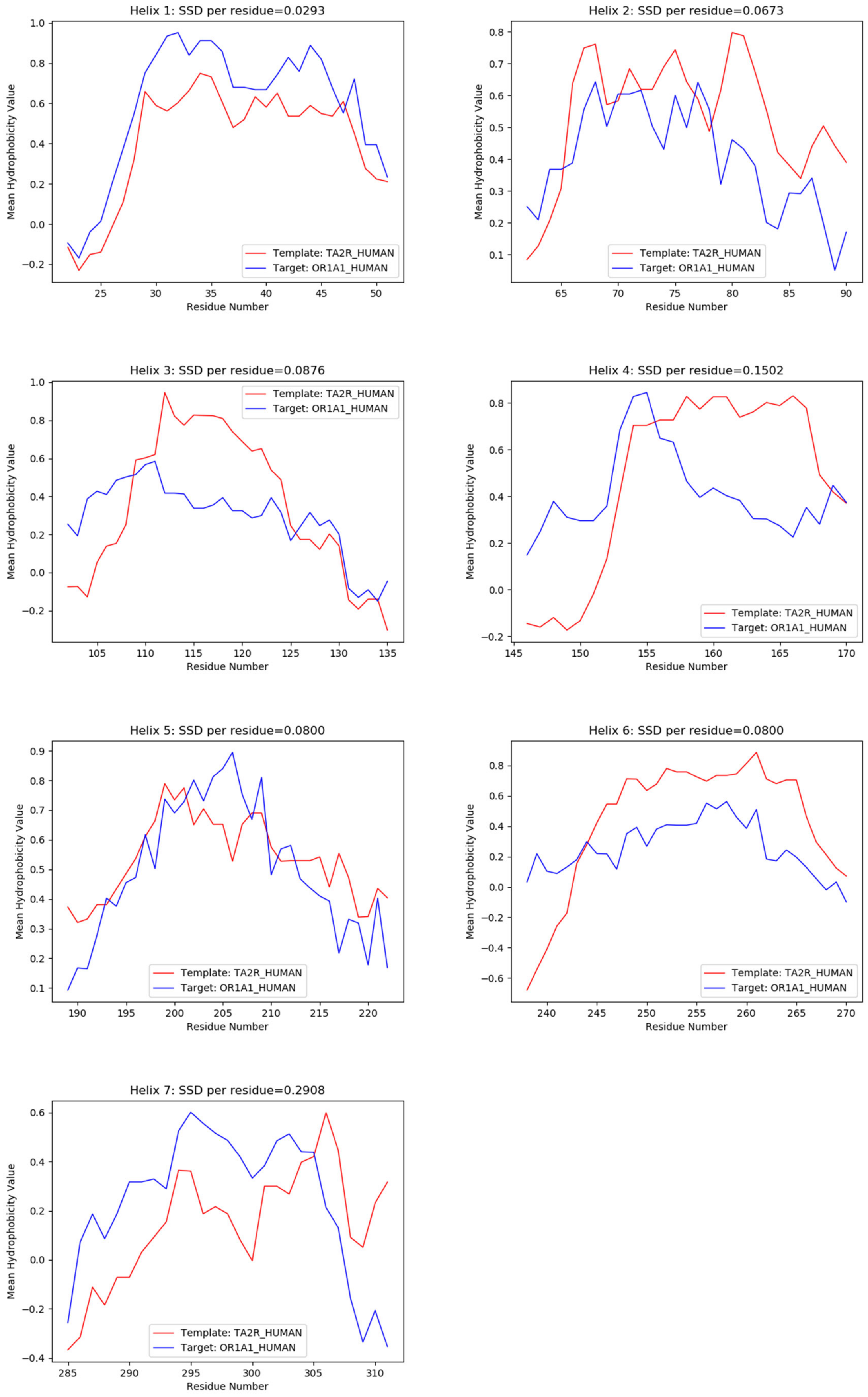

Supplementary Figure 5: **Helix-wise hydrophobicity correspondence between OR1A1 and 6IIU** (the 3rd best template).

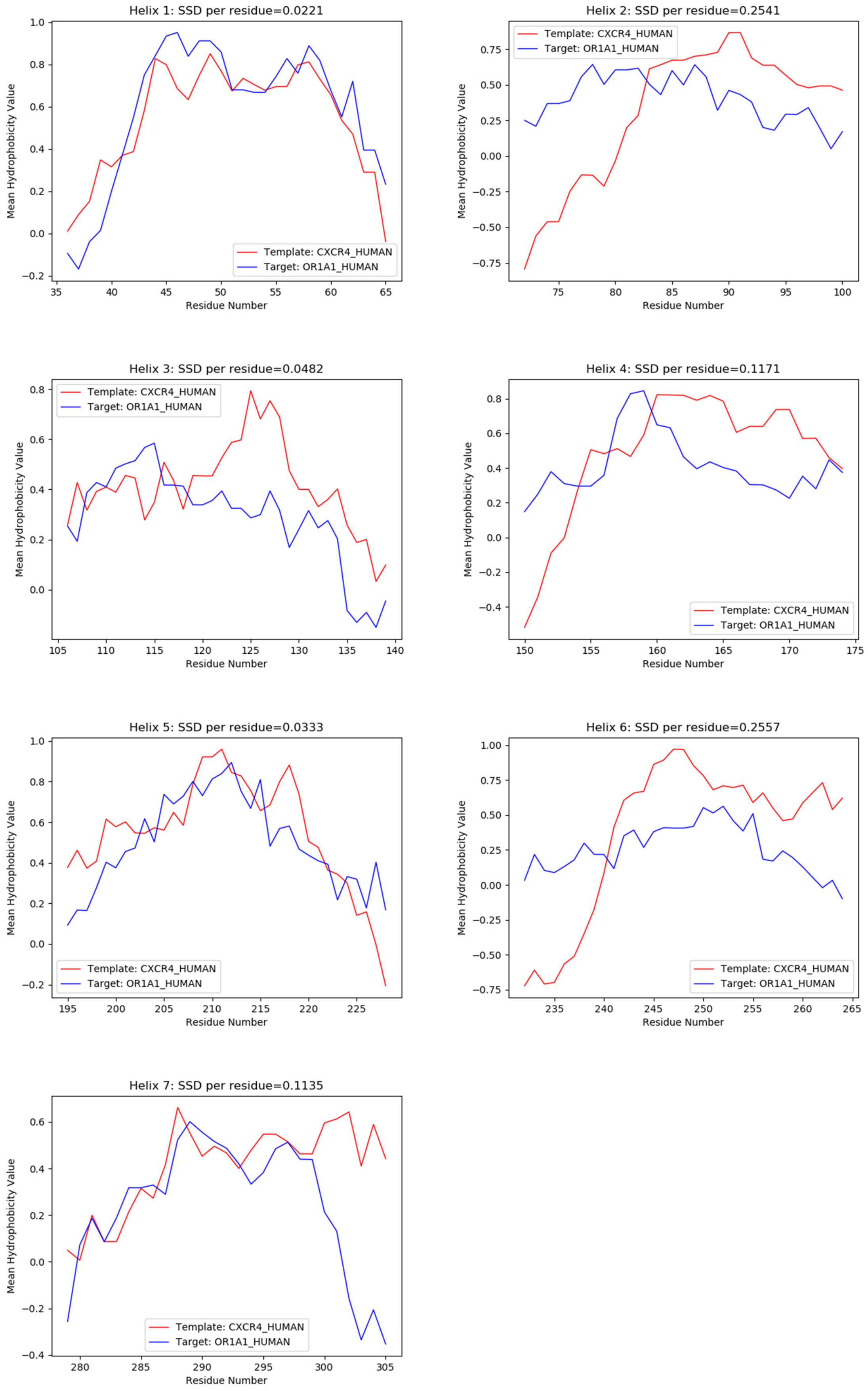

Supplementary Figure 6: Helix-wise hydrophobicity correspondence between OR1A1 and 3ODU (a template with low hydrophobicity correspondence).

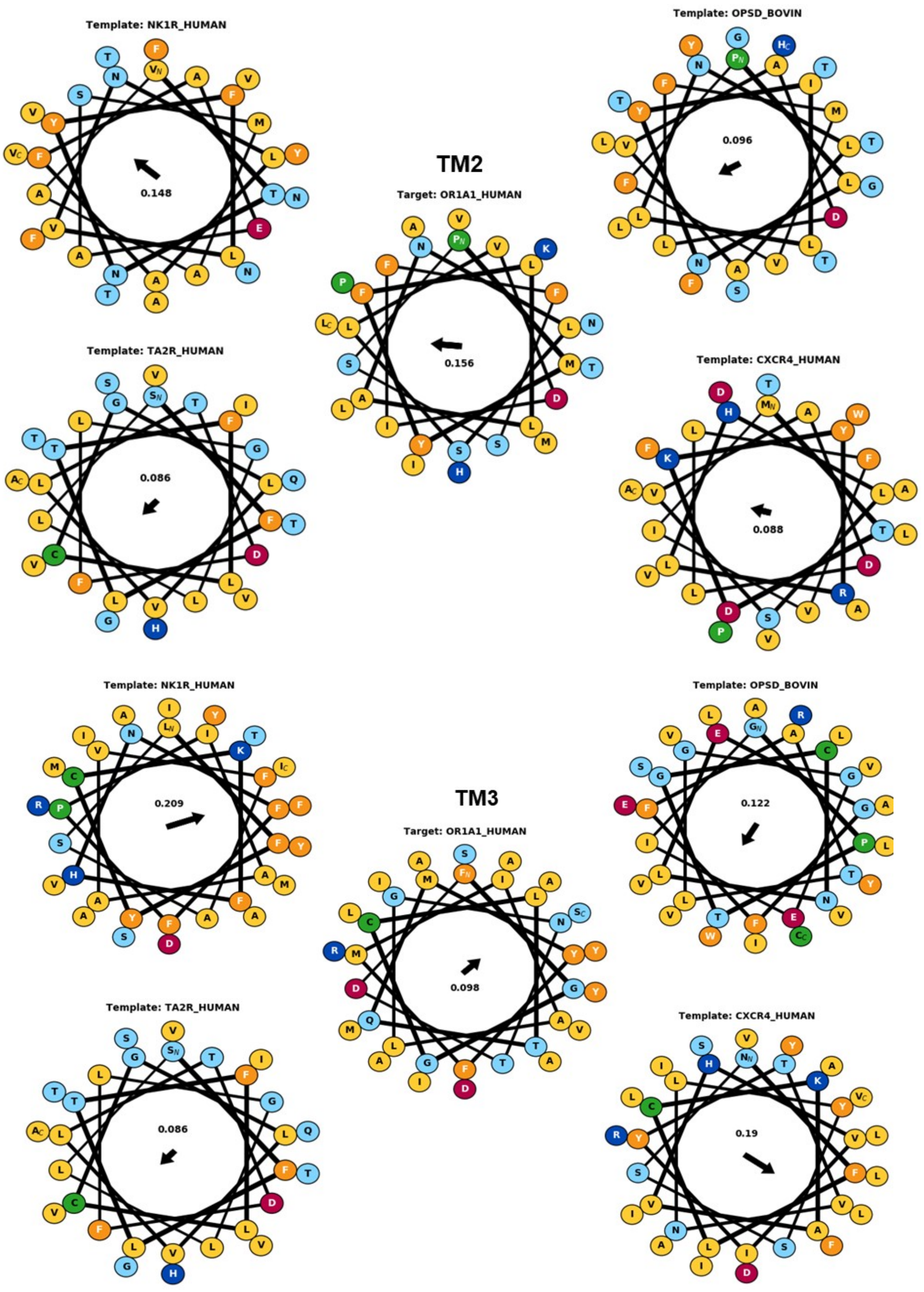

Supplementary Figure 7: **Helical wheel plots taken from Bio-GATS for TM2 and TM3** of target sequence (OR1A1) and the templates (NK1R_Human (6HLP), OPSD_BOVIN (1U19), TA2R_Human (6IIU), and CXCR4_Human (3ODU)). For TM3, the hydrophobic moment for OR1A1, 1U19, 6HLP, and 3ODU are pointing in almost same directions while for 6IIU, it is pointing in different directions. The hydrophobic moment for TM3 is pointing in different directions for all templates and the target.

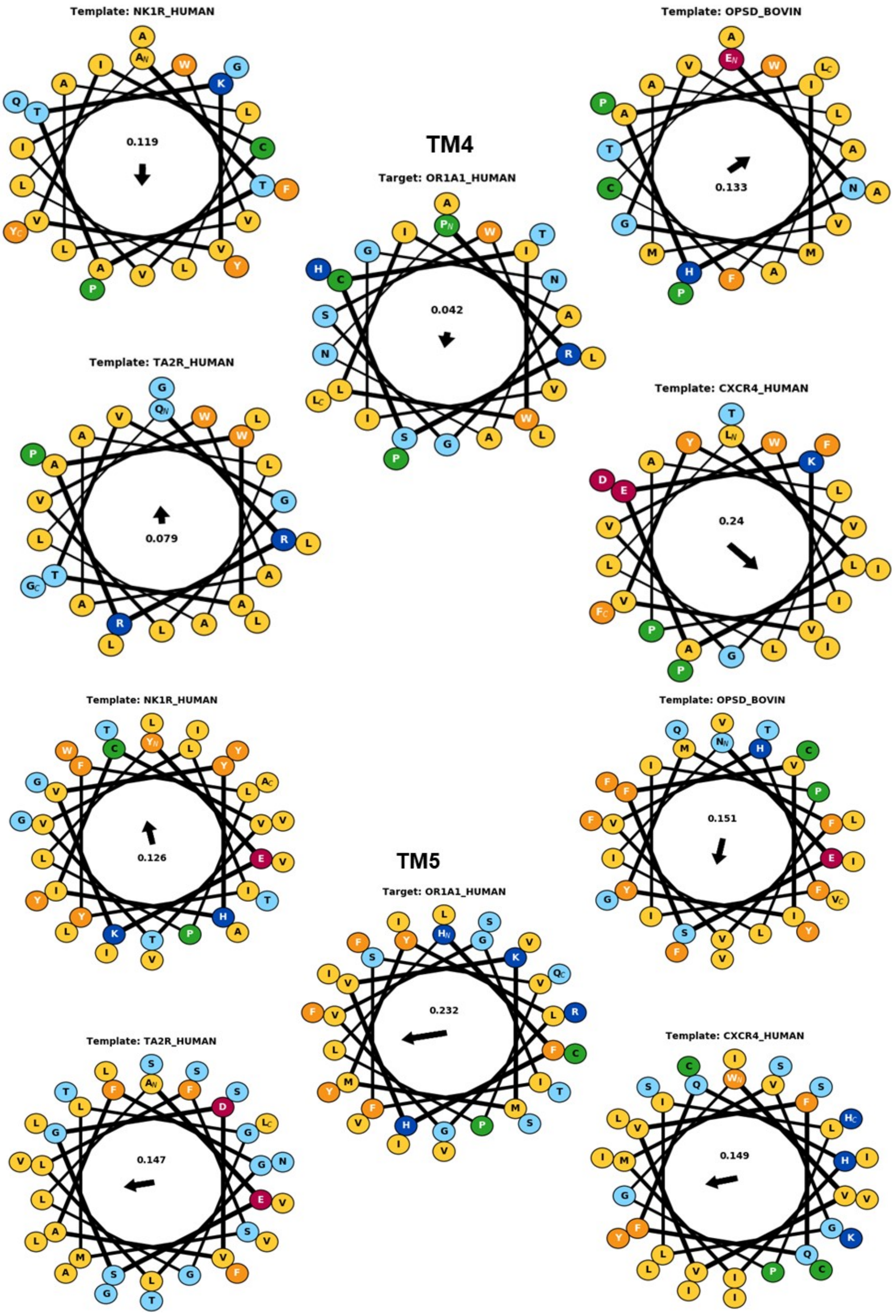

Supplementary Figure 8: **Helical wheel plots taken from Bio-GATS for TM4 and TM5** of target sequence (OR1A1) and the templates (NK1R_Human (6HLP), OPSD_BOVIN(1U19), TA2R_Human (6IIU), and CXCR4_Human (3ODU)). Within TM4, the hydrophobic moment for OR1A1 and 6HLP are pointing in same directions while for the other three it is pointing in different directions. The hydrophobic moment within TM5 for OR1A1 and all templates except 6HLP are pointing in same directions.

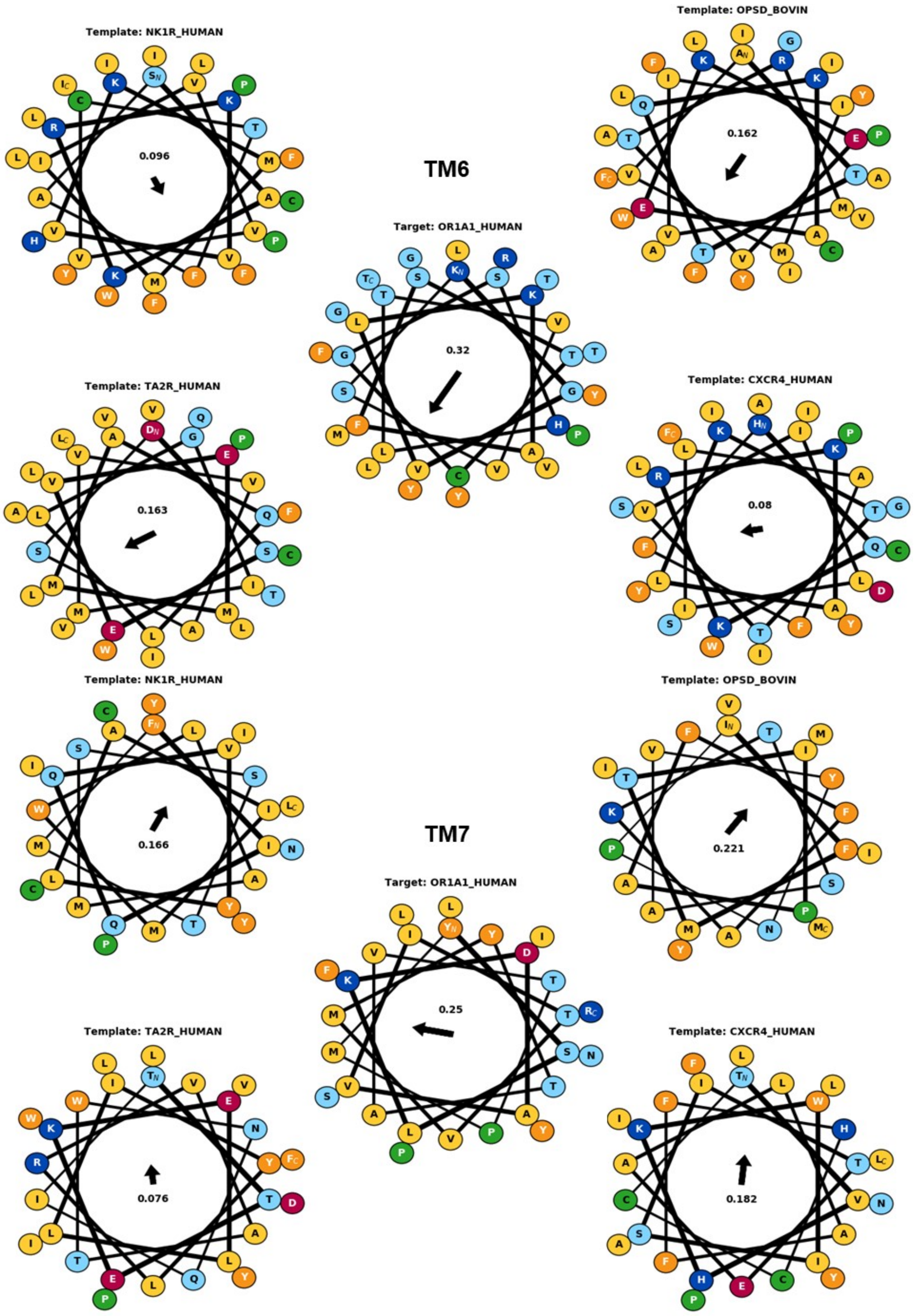

Supplementary Figure 9: **Helical wheel plots taken from Bio-GATS for TM6 and TM7** of target sequence (OR1A1) and the templates (NK1R_Human (6HLP), OPSD_BOVIN(1U19), TA2R_Human (6IIU), and CXCR4_Human (3ODU)). The hydrophobic moment within TM6 for OR1A1 and all templates except 6HLP are pointing in same directions. For TM7 no template is showing the moment pointing in the same direction as target.

Supplementary Note S1: **Bio-GATS result summary file for OPSD_BOVIN-OR1A1_HUMAN**

Output from OPSD_BOVIN-OR1A1_HUMAN

Please cite: Jabeen A., Ranganathan, S. BIO-GATS: A tool for automated GPCR template selection through a biophysical approach. Front. Mol. Biosci. 8:617176.

```
>OPSD_BOVIN
M--NG----T-EGPNFY----VPFSNKTGVVRSPFEAPQYYLAEPPWQFSMLAAYMFLLIMLGFPINFLTLYVTVQKKLR
T---PPLNYILLNLAVADLFMVFGGFTTTLYTSLHYF----V-FGGPTGCNLEGFFATLGGEIALWSLVVLAIERYVVVC
P------MSNFRFGE-ENHAIMGVAFTWVMALACAAPPL--GW-SRYIPEGMQCS-CG---I-DYY---TP---------
HEETNN-NESFVIYMFVVHFIIPLIVIFFCYGQLVFTV--EAAAQQQE-SA--ATTQKAEKEVTRMVIIMVIAFLICWLP
YAGVAFYIF-HQGSDFGPI---IFMTIPAFFAKTSAVYNPVIYIMMKQFRNCMVTT---LCCG-K--NPLGDDEASTT-V
SKTETSQVAPA
>OR1A1_HUMAN
MREN-NQSSTLE---F-ILLGV-----TG------Q--Q----E--EQEDFFYILFLFIYPITLIGNLLIVLAICS--VR
-LHNPPMYFLLANLSLVDIFFSSVTIPKMLANHL---GSKSISF--FGGCLTQMYFMIALGNTDSYILAAMAYDRAVAIS
PLHYTTIMS------PPRSCIWLIAGSWVIGNANALPHTLL--AS------L--SFCGNQEVANFYCDITPLLKLSCSDI
H-----HFHVKMMYLGVGIFSVPLLCIIVSYIRVFSTVFQ--------PS-TK----KGVLKAFSTCGSHLTVVSLYYGT
VMGTYFRPLT-----Y---YSLKDAVITVMYTAVTPMLNPFIYSLR---RD-M---KAAL---RKLFN-----K----RI
S----S-----
```

TM1

Template: PWQFSMLAAYMFLLIMLGFPINFLTLYVTVQ
Target: -EQEDFFYILFLFIYPITLIGNLLIVLAICS

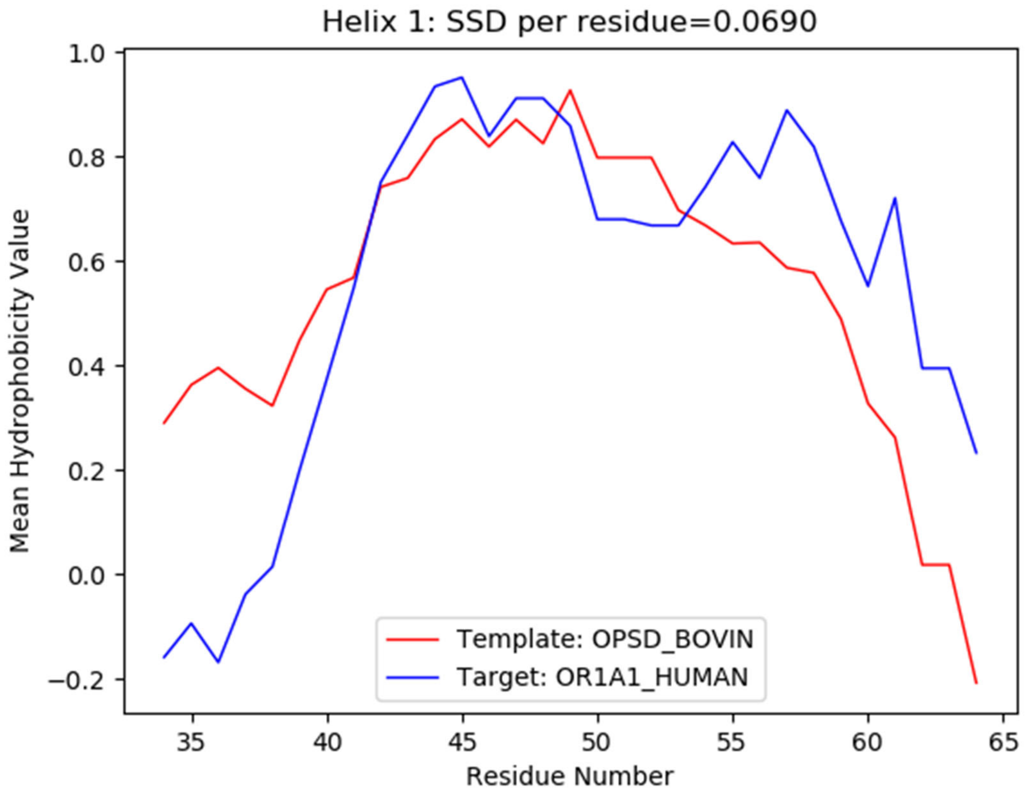

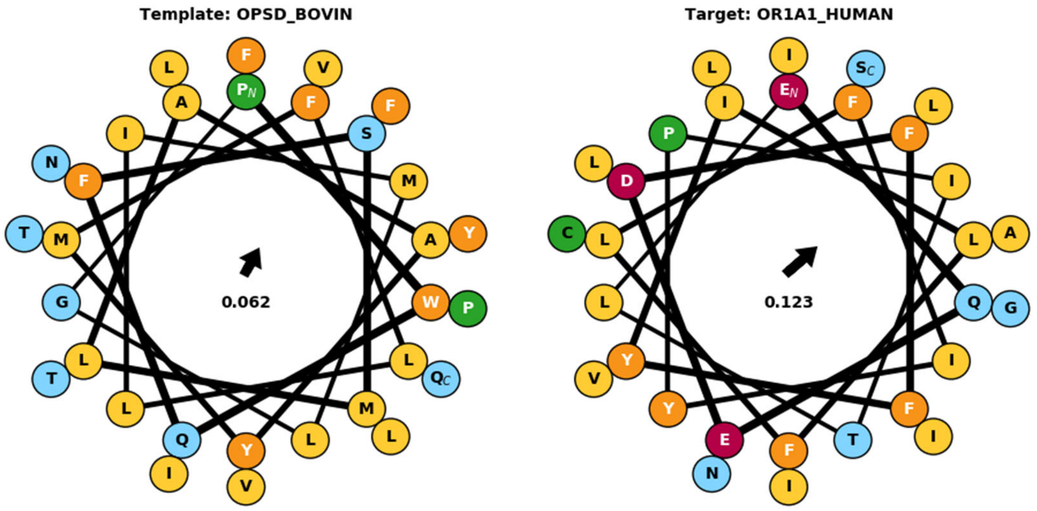

TM2

Template: PLNYILLNLAVADLFMVFGGFTTTLYTSLH
Target: PMYFLLANLSLVDIFFSSVTIPKMLANHL-

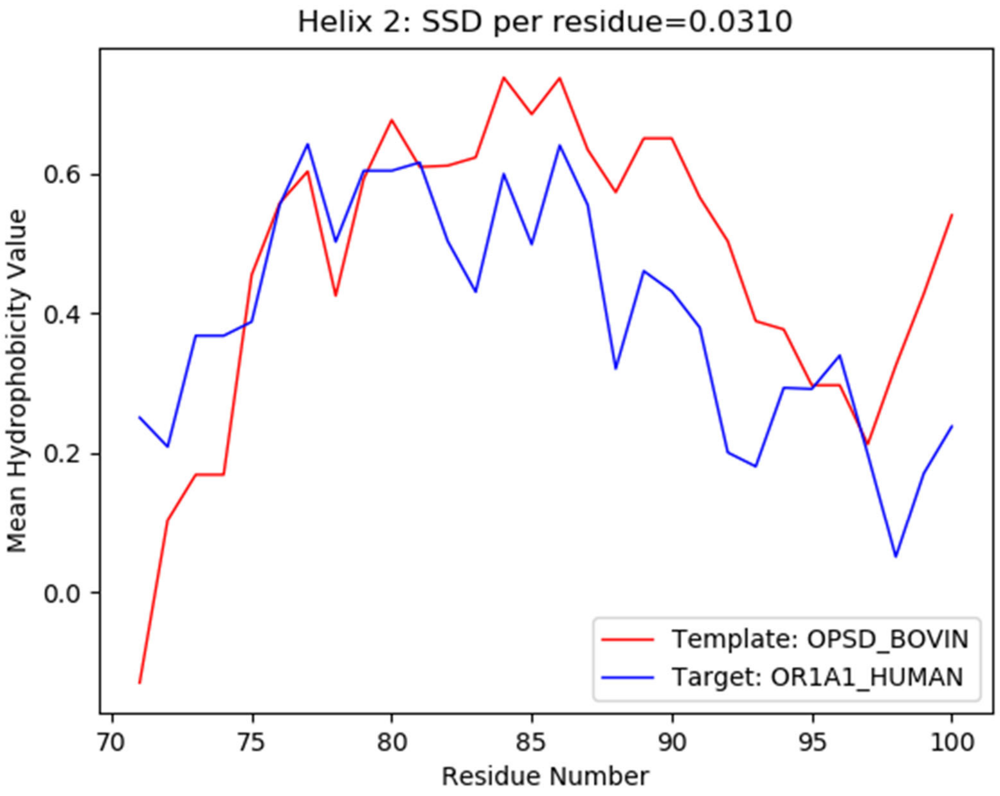

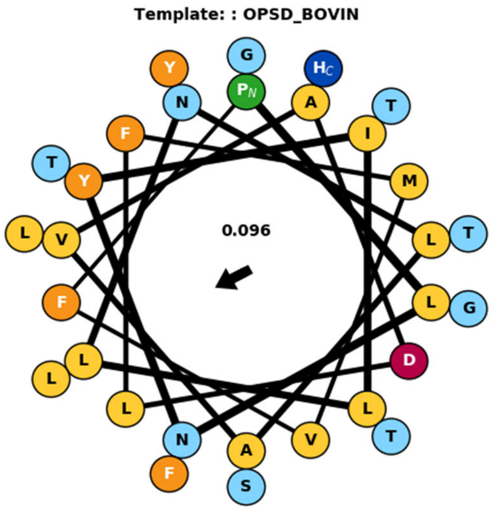

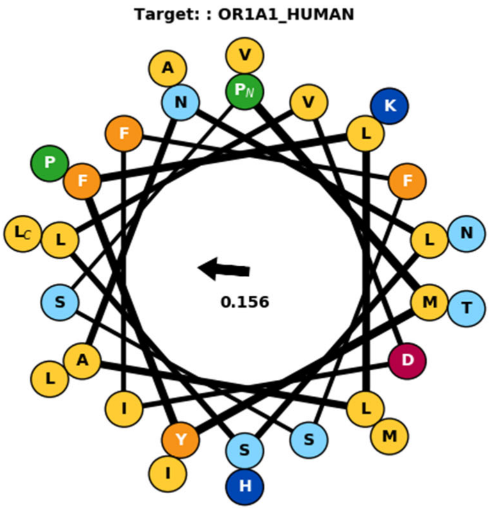

TM3

Template: GPTGCNLEGFFATLGGEIALWSLVVLAIERYVVVC
Target: -FGGCLTQMYFMIALGNTDSYILAAMAYDRAVAIS

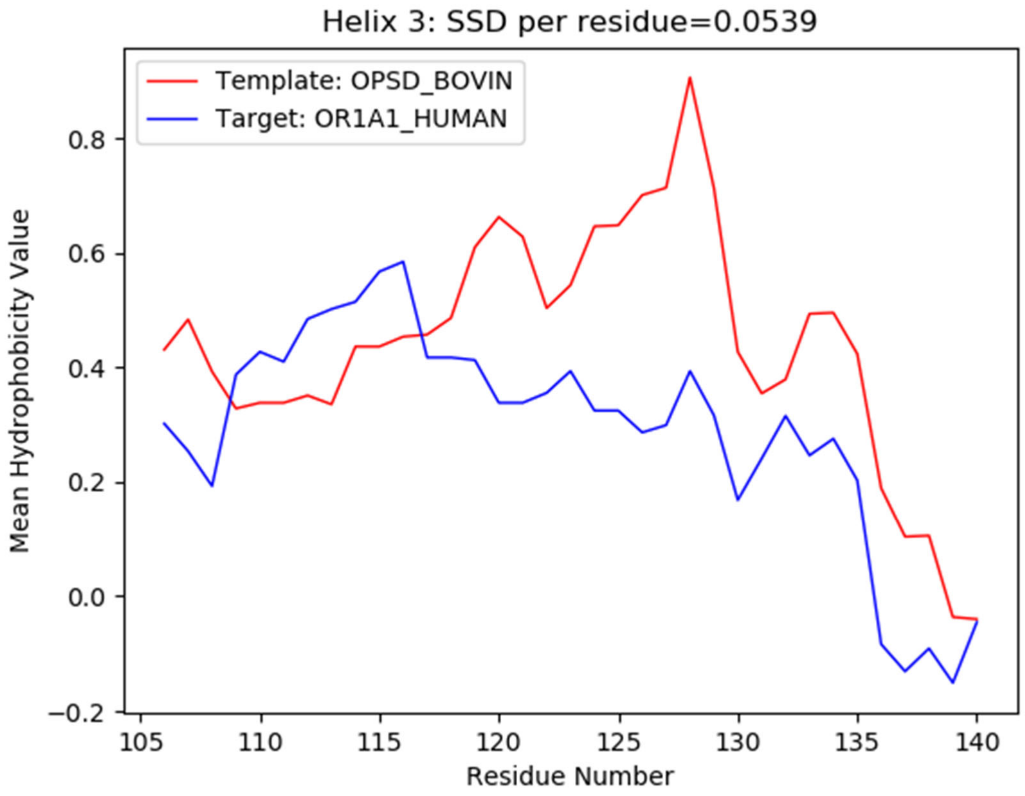

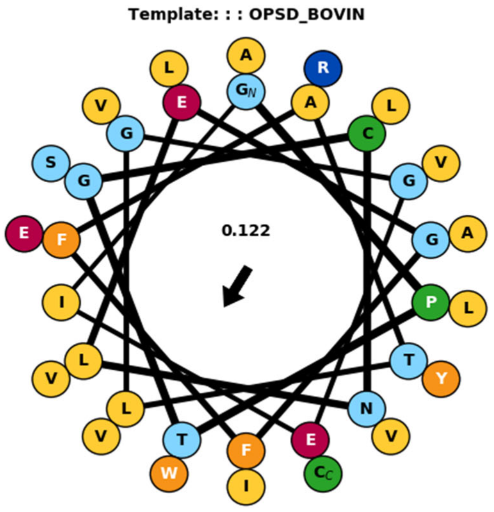

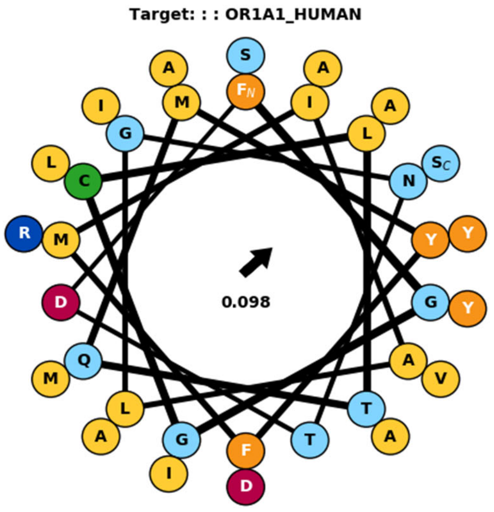

TM4

Template: ENHAIMGVAFTWVMALACAAPPL--
Target: PRSCIWLIAGSWVIGNANALPHTLL

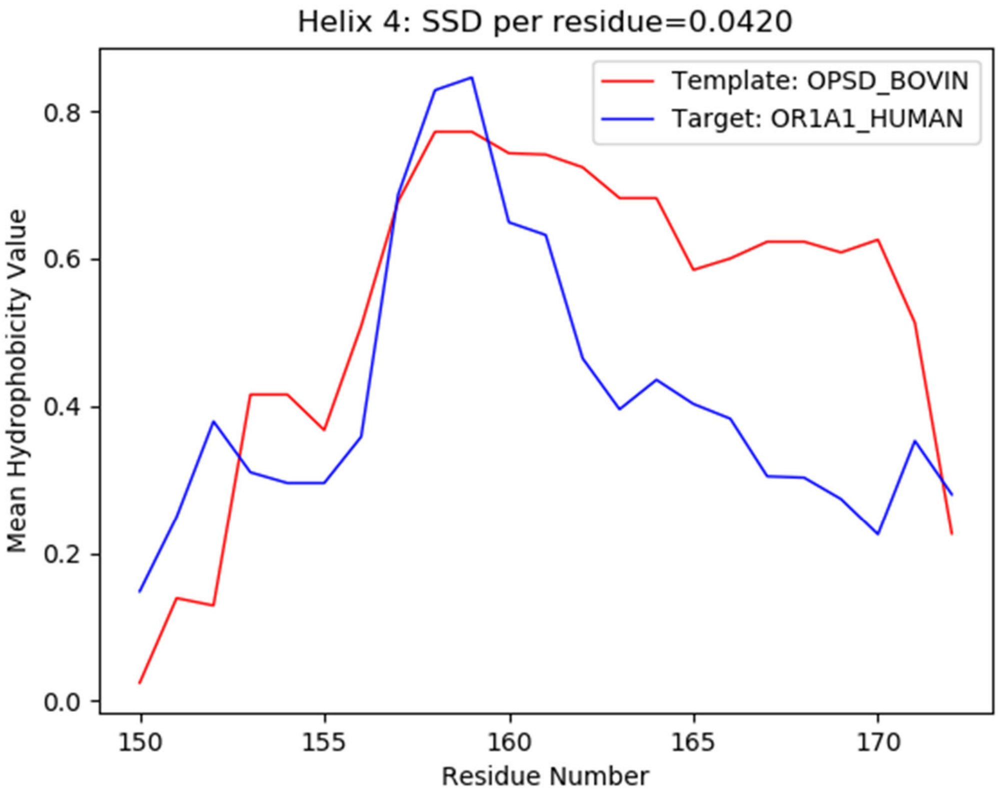

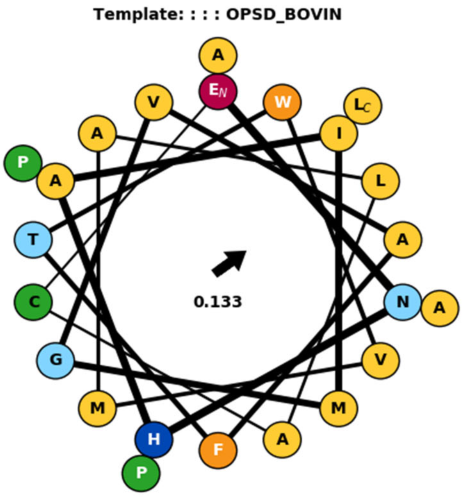

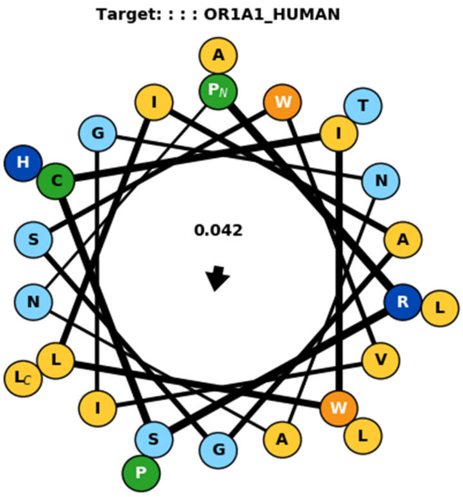

TM5

Template: -NESFVIYMFVVHFIIPLIVIFFCYGQLVFTV--
Target: HFHVKMMYLGVGIFSVPLLCIIVSYIRVFSTVFQ

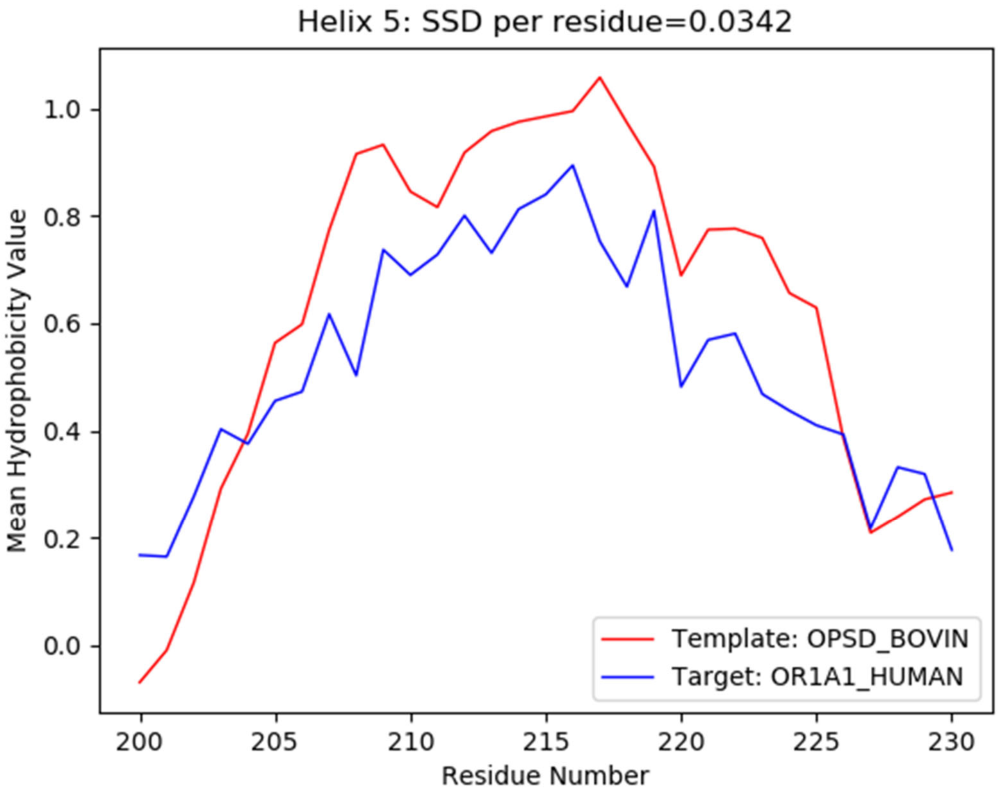

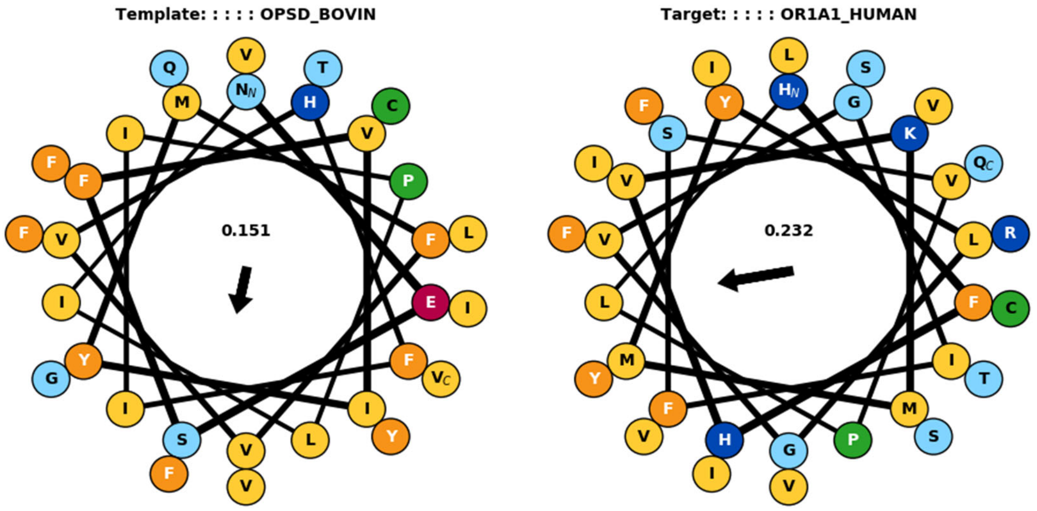

TM6

Template: ATTQKAEKEVTRMVIIMVIAFLICWLPYAGVAFYIF-
Target: ----KGVLKAFSTCGSHLTVVSLYYGTVMGTYFRPLT

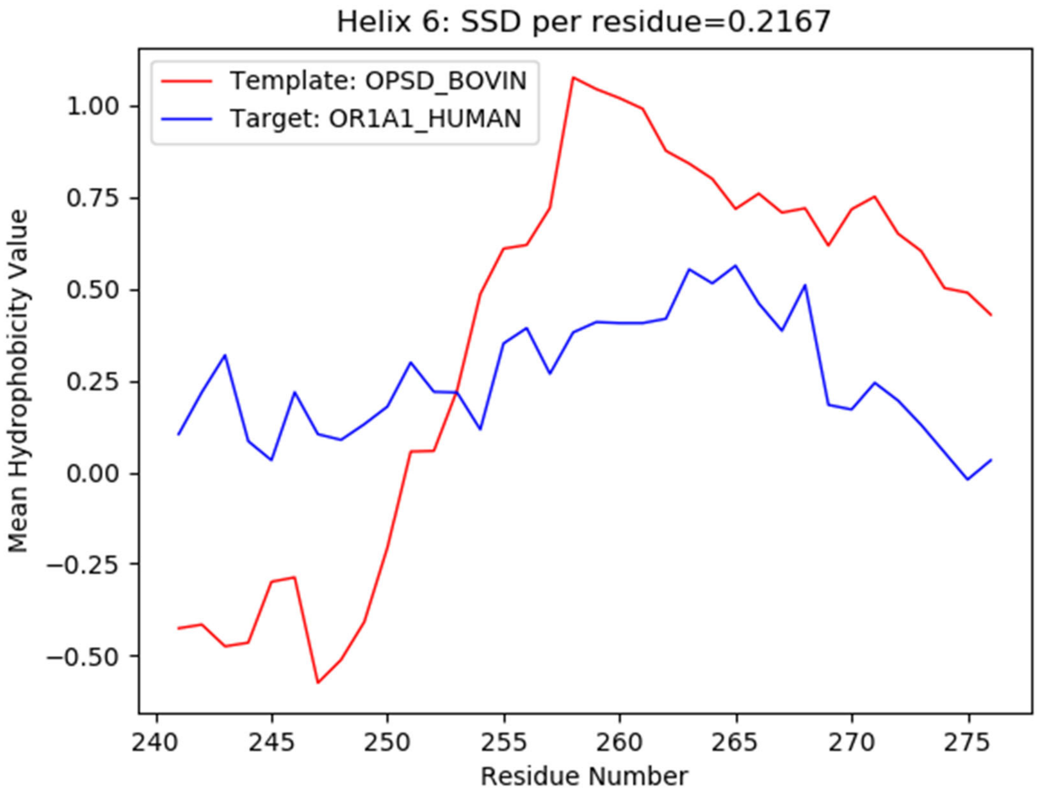

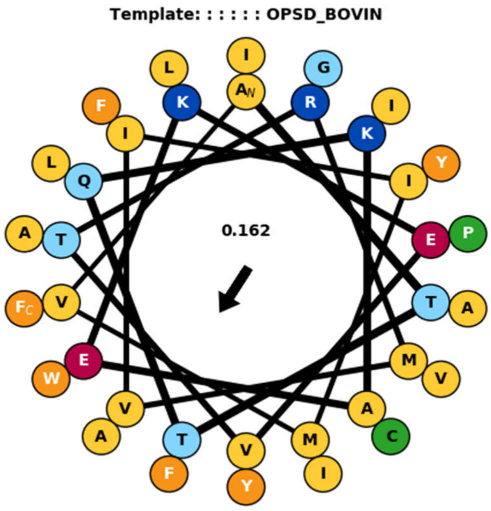

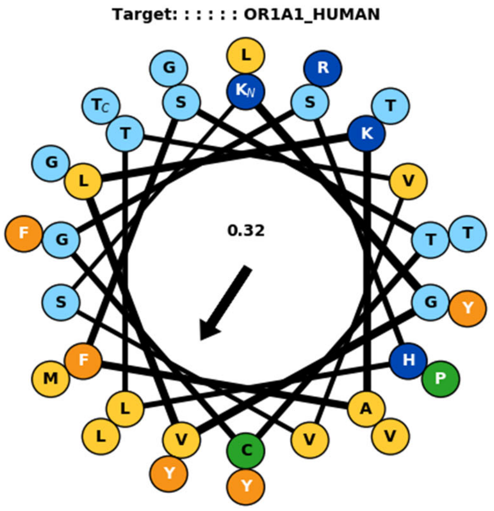

TM7

Template: ---IFMTIPAFFAKTSAVYNPVIYIMM
Target: YSLKDAVITVMYTAVTPMLNPFIYSLR

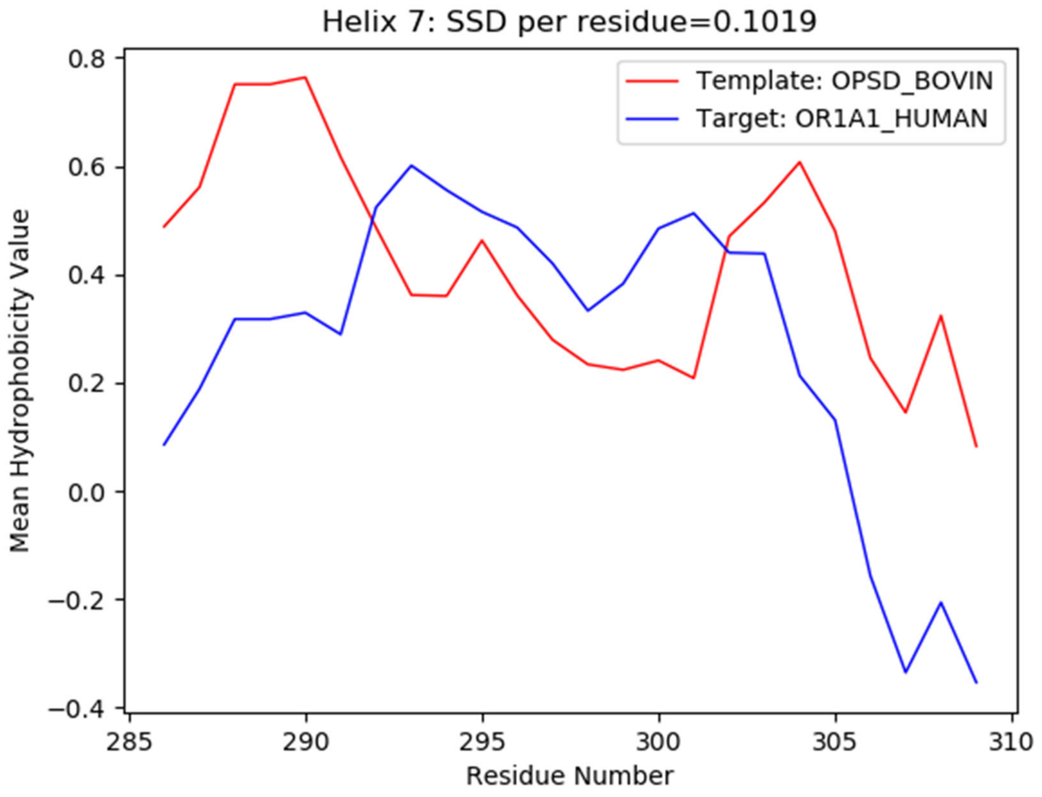

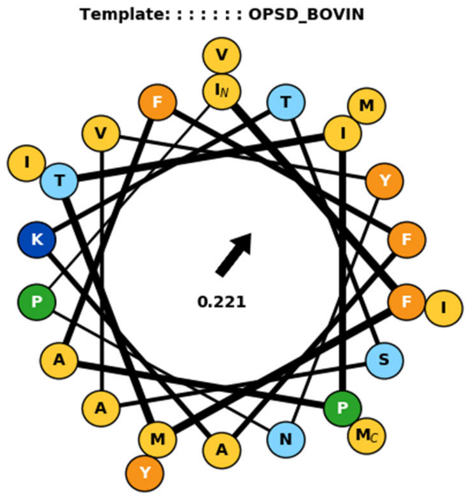

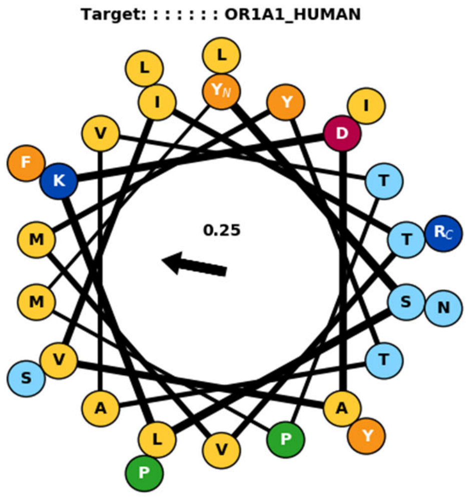

Supplementary Table 4: **Ligand profile comparison between OR1A1 and the selected templates**

No.	OR1A1 ligands	PubChem ID	No.	OR1A1 ligands	PubChem ID
1	(S)-(-)-citronellal	443157	31	Quinoline	7047
2	4-decenal	61875	32	R-limonene	440917
3	(S)-(-)-citronellol	7793	33	(R/S)-octen-3-ol	6992244
4	(R)-(+)-citronellol	75427	34	2-pentylpyridine	16800
5	(S)-(-)-limonene	439250	35	2-phenylethanethiol	78126
6	Z-7-decanal	5362695	36	2-Phenylethyl acetate	7654
7	Nerolidol	5284507	37	3-Mercaptohexyl acetate	518810
8	E-4-decanal	5702654	38	Estragole	8815
9	Nerol	643820	39	Ethyl cyclohexane-carboxylate	18686
10	Helional	64805	40	Trans-Anethole	637563
11	(-)-Carveol	11084068	41	3-Methyl-2,4-nonanedione	529481
12	Allyl heptanoate	8878	42	Ethylphenyl acetate	7590
13	Ethyl hexanoate	31265	43	5-Pentyloxolan-2-one	7710
14	(-)-Carvone	439570	44	(+)-Menthone	443159
15	(+)-Dihydrocarvone	22227	45	Musk xylol	62329
16	1-decanol	8174	46	Cosmone	66823518
17	2-octanone	8093	47	Celestolide	61585
18	3-heptanone	7802	48	2-ethylphenol	6997
18	3-octanone	246728	49	Methyl isoeugenol	7128
20	4-chromanone	68110	50	P-Tolyl isobutyrate	7685
21	Allyl phenylacetate	15717	51	Propiophenone	7148
22	Benzophenone	3102			
23	Benzyl acetate	8785			
24	Dihydrojasmone	62378			
25	Nonanethiol	15077			
26	(+)-Carvone	16724			
27	3-phenyl propyl propionate	61052			
28	Androstadienone	92979			
29	Butyl anthranilate	24433			
30	Cinnamaldehyde	637511			

Supplementary Table 5: **The interactions of 1U19-based and 3ODU-based OR1A1 models with known ligands of OR1A1 with mutagenesis data.** Hydrophobic regions in green, van der Waals interactions in grey surface accessible regions in grey parabolas; hydrogen bond acceptors in blue. The residues having mutagenesis data are boxed in red.

<table>
<tr><th>1U19-based OR1A1 model</th><th>3ODU-based OR1A1 model</th></tr>
<tr><td colspan="2">(*S*)-(-) –citronellol</td></tr>
<tr><td>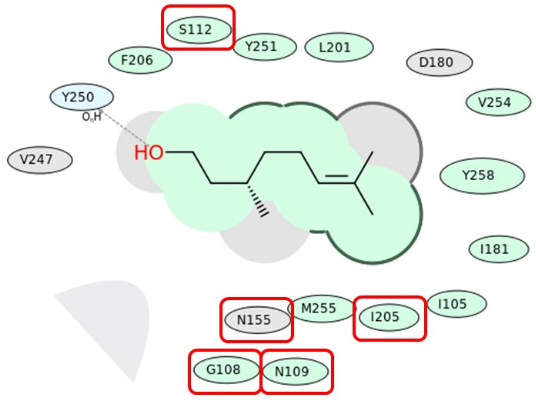
</td><td>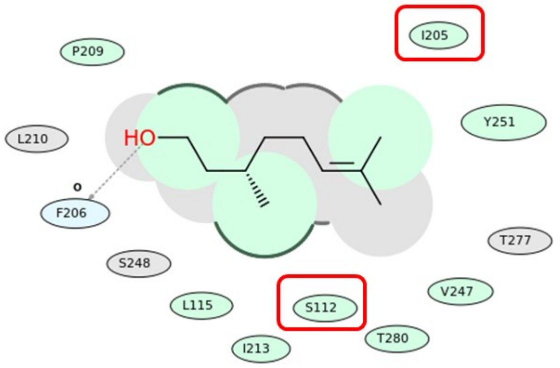
</td></tr>
<tr><td colspan="2">(*S*)- (-) –citronellal</td></tr>
<tr><td>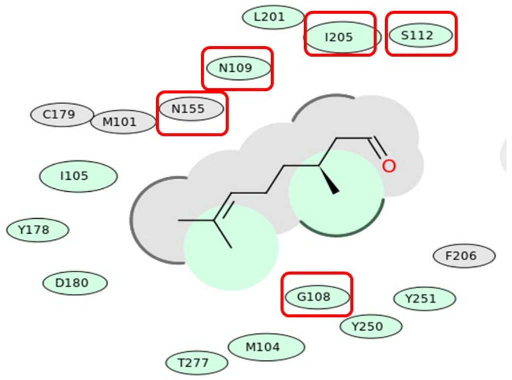
</td><td>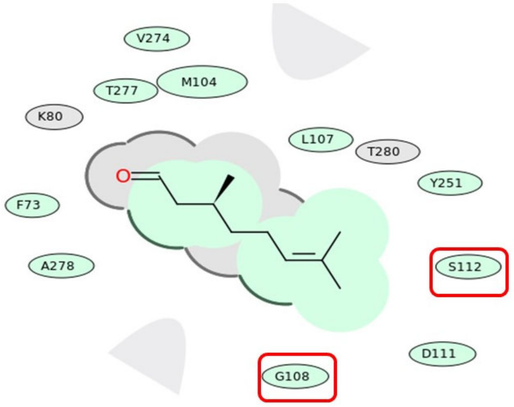
</td></tr>
<tr><td colspan="2">(*S*)- (+)-carvone</td></tr>
<tr><td>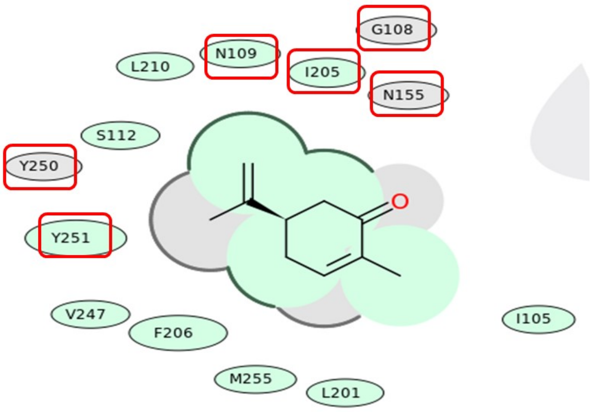
</td><td>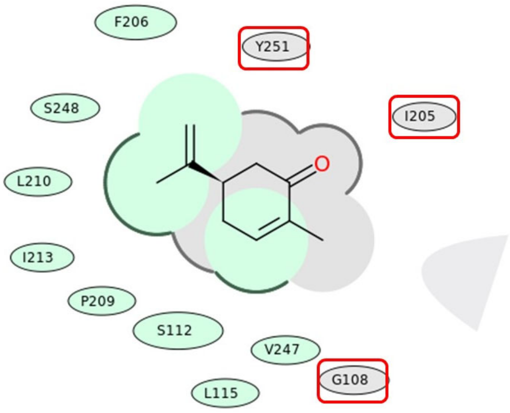
</td></tr>
</table>

<table>
<tr><th>1U19-based OR1A1 model</th><th>3ODU-based OR1A1 model</th></tr>
<tr><td colspan="2">(R)- (-) –carvone</td></tr>
<tr><td>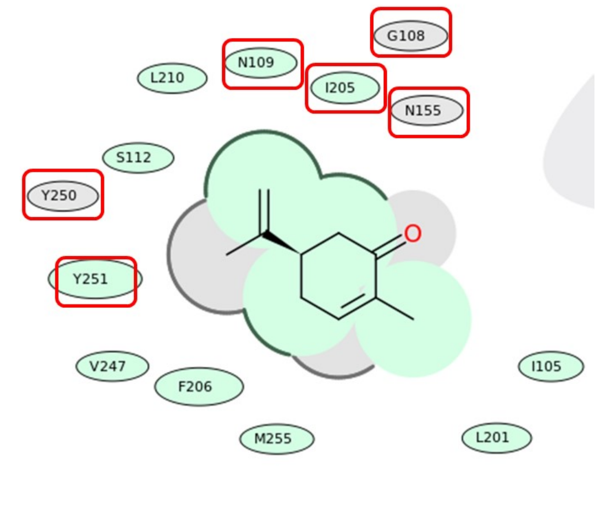
</td><td>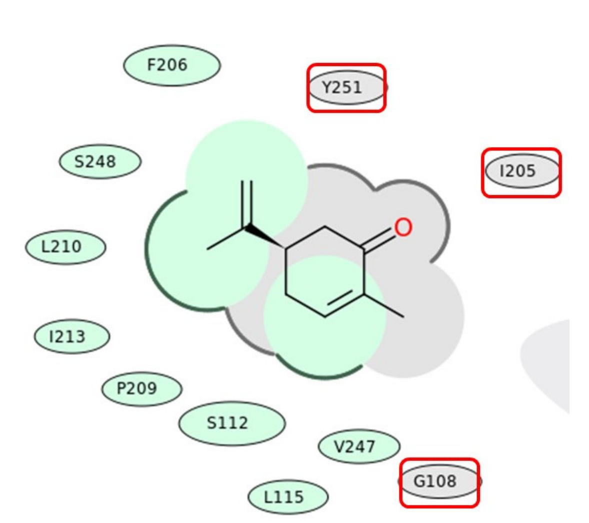
</td></tr>
<tr><td colspan="2">Musk tibetene</td></tr>
<tr><td>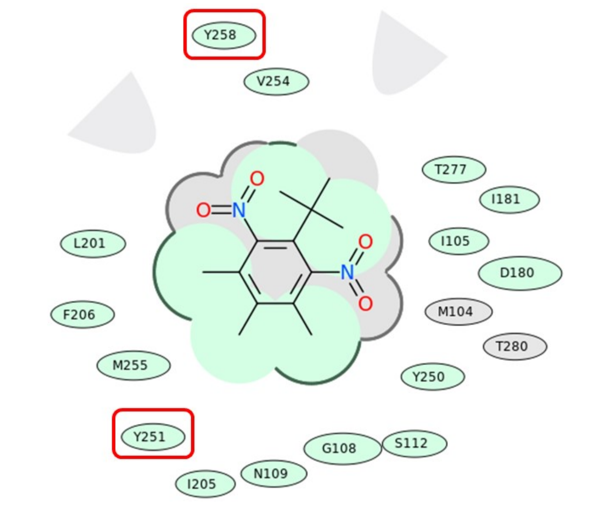
</td><td>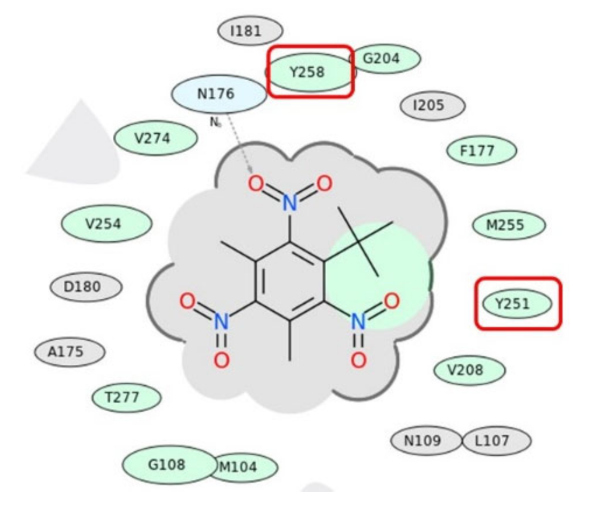
</td></tr>
<tr><td colspan="2">Musk xylene</td></tr>
<tr><td>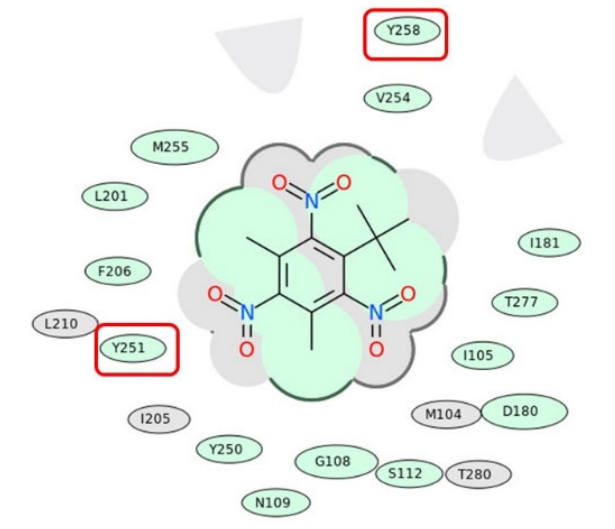
</td><td>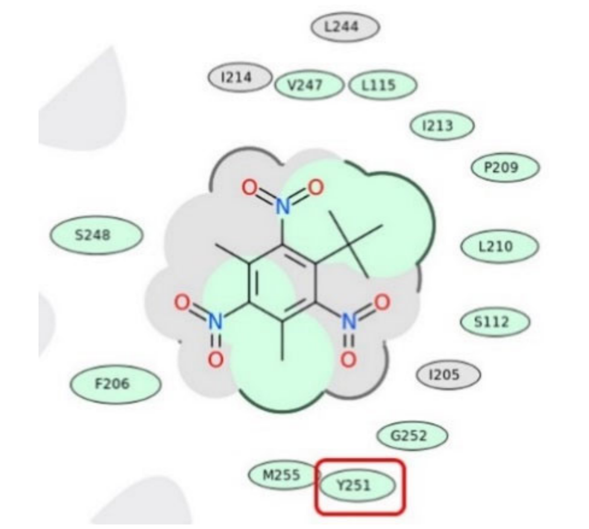
</td></tr>
</table>

```
                                                                                 1.50
3EML_A    -------------IMGSS-------VYITVELAIAVLAILGNVLVCWAVWLNSNLQNVTN 40
OR1A1     MRENNQSSTLEFILLGVTGQQEQEDFFYILFLFIYPITLIGNLLIVLAICSDVRLHNPMY 60
                    2.50
3EML_A    YFVVSLAAADIAVGVLAIP--FAITISTGFCAACHGCLFIACFVLVLTQSSIFSLLAIAI 98
OR1A1     FLLANLSLVDIFFSSVTIPKMLANHLLGSKSISFGGCLTQMYFMIALGNTDSYILAAMAY 120
           3.50                        4.50
3EML_A    DRYIAIRIPLRYNGLVTGTRAKGIIAICWVLSFAIGLTPMLGWNNCGQSQGCGEGQVACL 158
OR1A1     DRAVAISRPLHYTTIMSPRSCIWLIAGSWVIGNANALPHTLLTASLS---FCGNQEVANF 177
              5.50
3EML_A    FEDVVPMNYMVYFNFFACVLVPLL---LMLGVYLRIFLAARRQLRSTLQKEVHAAKSLAI 215
OR1A1     YCDITPLLKLSCSDIHFHVKMMYLGVGIFSVPLLCIIVSYIRVFSTVFQVPSTKGVLKAF 237
                    6.50                                        7.50
3EML_A    IVGLFALCWLPLHIINCFTFFCPDCSHAPLWLMYLAIVLSHTNSVVNPFIYAYRIREFRQ 275
OR1A1     STCGSHLTVVSLYYGTVMGTYFRPLTNYSLKDAVITVMYTAVTPMLNPFIYSLRNRDMKA 297

3EML_A    TFRKIIRSHVLRQ 288
OR1A1     ALRKLFNKRISS- 309
```

Supplementary Figure 10: **The alignment generated by GPCR-I-TASSER between query sequence (OR1A1) and the top template (3EML).** The center TM residues in each sequence are in red colour.

```
                                                                                 1.50
3EML      --------------------IMGSSVYITVELAIAVLAILGNVLVCWAVWLNSNLQNVTN 40
OR1A1     MRENNQSSTLEFILLGVTGQQEQEDFFYILFLFIYPITLIGNLLIVLAICSDVRLHNPMY 60

                    2.50
3EML      YFVVSLAAADIAVGVLAIPFAITISTG--FCAACHGCLFIACFVLVLTQSSIFSLLAIAI 98
OR1A1     FLLANLSLVDIFFSSVTIPKMLANHLLGSKSISFGGCLTQMYFMIALGNTDSYILAAMAY 120

          3.50                         4.50
3EML      DRYIAIRIPLRYNGLVTGTRAKGIIAICWVLSFAIGLTPMLGWNNCG-----QSQGCGEG 153
OR1A1     DRAVAISRPLHYTTIMSPRSCIWLIAGSWVIGNANAL-PHTLLTASLSFCGNQEVANFYC 179

                                        5.50
3EML      QVACLFEDVVPMNYMV---YFNFFACVLVPLLLMLGVYLRIFLAARRQLRSTLQKEVHAA 210
OR1A1     DITPLLKLSCSDIHFHVKMMYLGVGIFSVPLLCIIVSYIRVFSTVFQ------------V 227

                    6.50                                         1.50
3EML      KSLAIIVGLFALCWLPLHIINCFTFF------CPDCSHAPLWLMYLAIVLSHTNSVVNPF 264
OR1A1     PSTKGVLKAFSTCGSHLTVVSLYYGTVMGTYF-RPLTNYSLKDAVITVMYTAVTPMLNPF 286

3EML      IYAYRIREFRQTFRKIIRSHVLRQ  288
OR1A1     IYSLRNRDMKAALRKLFNKRISS-  309
```

Supplementary Figure 11: **The alignment generated by GPCRM between query sequence (OR1A1) and the top template (3EML).** The center TM residues in each sequence are in red colour.

```
OPSD_BOVIN      M--NG----T-EGPNFY----VPFSNKTGVVRSPFEAPQYYLAEPPWQFSMLAAYMFLLI 39
OR1A1_HUMAN     MREN-NQSSTLE---F-ILLGV-----TG------Q--Q----E--EQEDFFYILFLFIY 35
                     1.50                           2.50
OPSD_BOVIN      MLGFPINFLTLYVTVQKKLRT---PPLNYILLNLAVADLFMVFGGFTTTLYTSLHYF--- 93
OR1A1_HUMAN     PITLIGNLLIVLAICS--VR-LHNPPMYFLLANLSLVDIFFSSVTIPKMLANHL---GSK 90
                                                 3.50
OPSD_BOVIN      -V-FGGPTGCNLEGFFATLGGEIALWSLVVLAIERYVVVCP------MSNFRFGE-ENHA 144
OR1A1_HUMAN     SISF--FGGCLTQMYFMIALGNTDSYILAAMAYDRAVAISPLHYTTIMS------PPRSC 141
                       4.50
OPSD_BOVIN      IMGVAFTWVMALACAAPPL--GW-SRYIPEGMQCS-CG---I-DYY---TP--------- 184
OR1A1_HUMAN     IWLIAGSWVIGNANALPHTLL--AS------L--SFCGNQEVANFYCDITPLLKLSCSDI 192
                                     5.50
OPSD_BOVIN      HEETNN-NESFVIYMFVVHFIIPLIVIFFCYGQLVFTV--EAAAQQQE-SA--ATTQKAE 238
OR1A1_HUMAN     H-----HFHVKMMYLGVGIFSVPLLCIIVSYIRVFSTVFQ--------PS-TK----KGV 233
                                  6.50                                    7.50
OPSD_BOVIN      KEVTRMVIIMVIAFLICWLPYAGVAFYIF-HQGSDFGPI---IFMTIPAFFAKTSAVYNP 294
OR1A1_HUMAN     LKAFSTCGSHLTVVSLYYGTVMGTYFRPLT-----Y---YSLKDAVITVMYTAVTPMLNP 285

OPSD_BOVIN      VIYIMMKQFRNCMVTT---LCCG-K--NPLGDDEASTT-VSKTETSQVAPA  338
OR1A1_HUMAN     FIYSLR---RD-M---KAAL---RKLFN-----K----RIS----S-----  309
```

Supplementary Figure 12: **The alignment generated by BIO-GATS between the query sequence (OR1A1) and the selected template, 1U19.** The center TM residues in each sequence are in red colour.

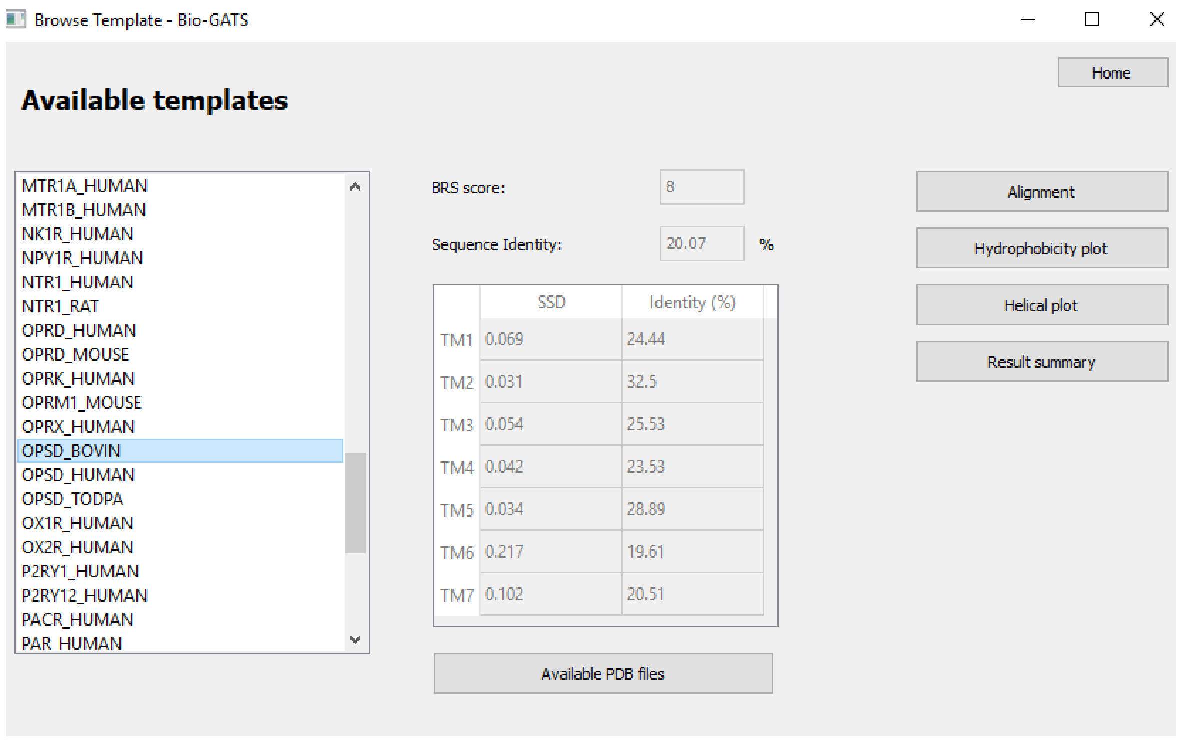

Supplementary Figure 13: **The *Browse template* window with options** for showing alignment, downloading hydrophobicity plots, helical wheel plots, and viewing available PDB data for each receptor. HC in terms of SSD, global sequence identity, TM-wise sequence identity, and binding site residue similarity (BRS) score are computed upon receptor selection.

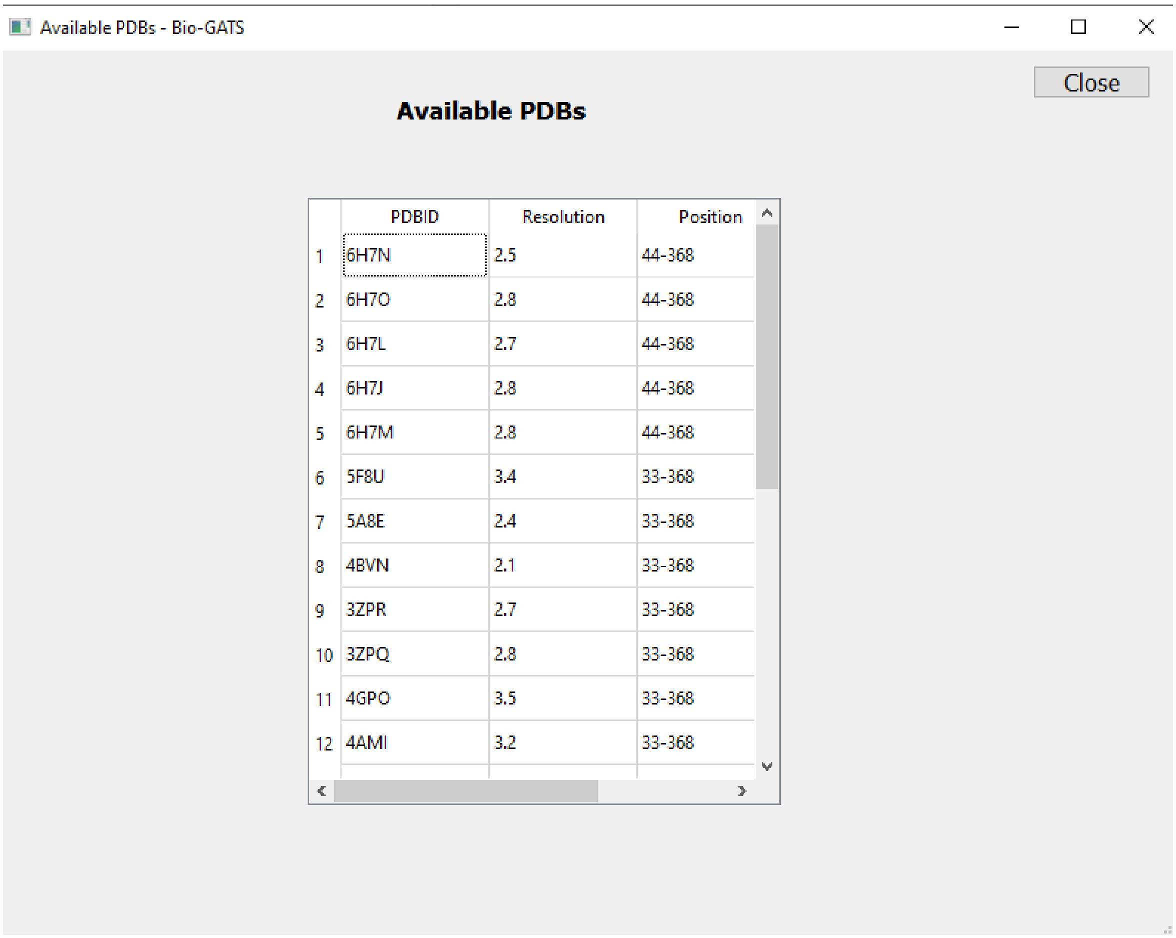

Supplementary Figure 14: **The *Available PDBs* window**, showing the PDB data for OPSD_MELGA

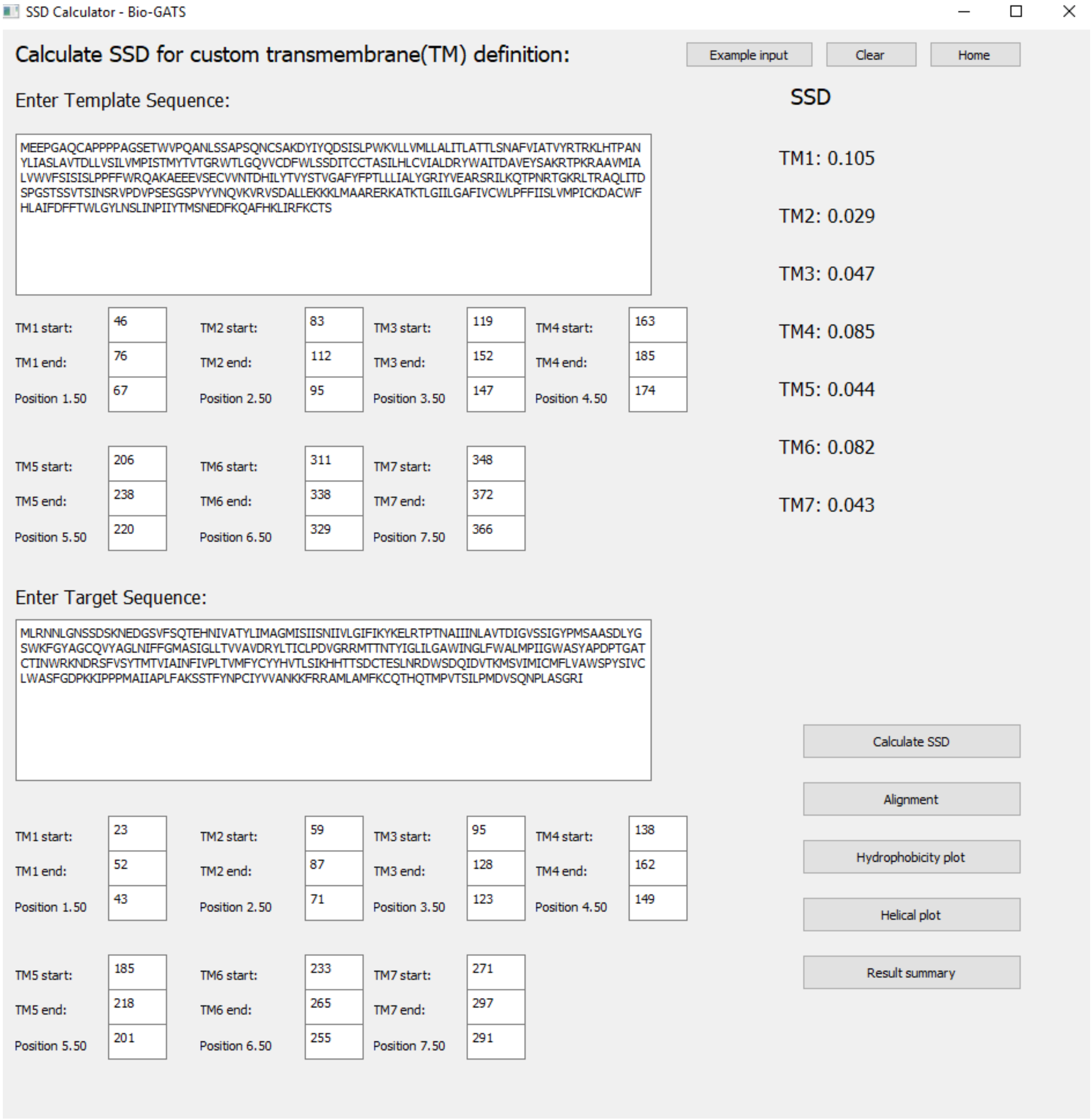

Supplementary Figure 15: **The *SSD calculator*** with customizable TM definitions.

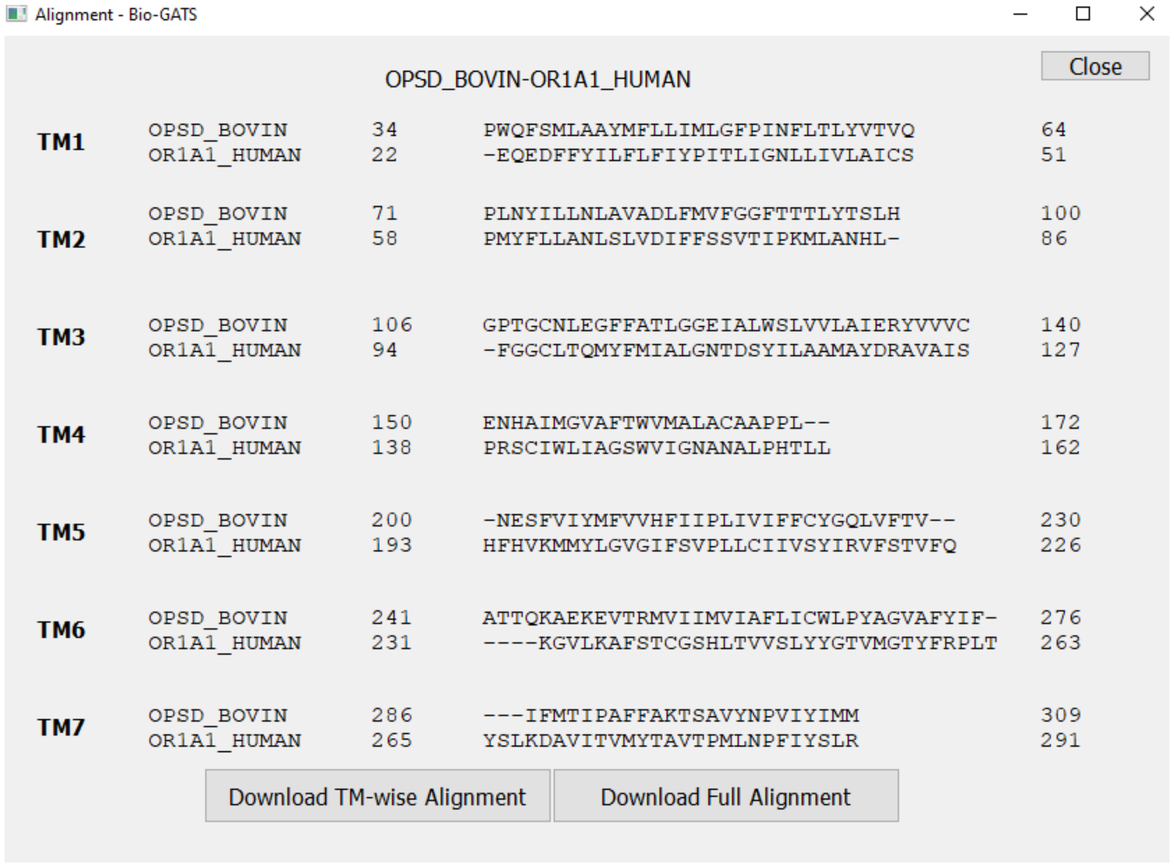

Supplementary Figure 16: **The *Show alignment* window** displaying the helix-wise alignment between OPSD_BOVINE and OR1A1

Supplementary References

1. Perry, S.R., W. Xu, A. Wirija, J. Lim, M.K. Yau, M.J. Stoermer, A.J. Lucke, and D.P. Fairlie, *Three Homology Models of PAR2 Derived from Different Templates: Application to Antagonist Discovery.* J Chem Inf Model, 2015. **55**(6): p. 1181-91.
2. Shahaf, N., M. Pappalardo, L. Basile, S. Guccione, and A. Rayan, *How to Choose the Suitable Template for Homology Modelling of GPCRs: 5-HT7 Receptor as a Test Case.* Mol Inform, 2016. **35**(8-9): p. 414-23.
3. Loo, J.S., A.L. Emtage, K.W. Ng, A.S. Yong, and S.W. Doughty, *Assessing GPCR homology models constructed from templates of various transmembrane sequence identities: Binding mode prediction and docking enrichment.* J Mol Graph Model, 2018. **80**: p. 38-47.
4. Castleman, P.N., C.K. Sears, J.A. Cole, D.L. Baker, and A.L. Parrill, *GPCR homology model template selection benchmarking: Global versus local similarity measures.* J Mol Graph Model, 2019. **86**: p. 235-246.
5. Jaiteh, M., I. Rodríguez-Espigares, J. Selent, and J. Carlsson, *Performance of virtual screening against GPCR homology models: Impact of template selection and treatment of binding site plasticity.* PLoS Comput Biol, 2020. **16**(3): p. e1007680.
